# Regulation of ICAM-1 in Cells of the Monocyte/Macrophage System in Microgravity

**DOI:** 10.1155/2015/538786

**Published:** 2015-01-13

**Authors:** Katrin Paulsen, Svantje Tauber, Claudia Dumrese, Gesine Bradacs, Dana M. Simmet, Nadine Gölz, Swantje Hauschild, Christiane Raig, Stephanie Engeli, Annett Gutewort, Eva Hürlimann, Josefine Biskup, Felix Unverdorben, Gabriela Rieder, Daniel Hofmänner, Lisa Mutschler, Sonja Krammer, Isabell Buttron, Claudia Philpot, Andreas Huge, Hartwin Lier, Ines Barz, Frank Engelmann, Liliana E. Layer, Cora S. Thiel, Oliver Ullrich

**Affiliations:** ^1^Institute of Anatomy, Faculty of Medicine, University of Zurich, Winterthurerstraß 190, 8057 Zurich, Switzerland; ^2^Department of Machine Design, Engineering Design and Product Development, Institute of Mechanical Engineering, Otto-von-Guericke-University Magdeburg, Universitätsplatz 2, 39106 Magdeburg, Germany; ^3^Flow Cytometry Facility, University of Zurich, Winterthurerstraß 190, 8057 Zurich, Switzerland; ^4^German Aerospace Center, Space Agency, Königswinterer Straße 522-524, 53227 Bonn, Germany; ^5^Integrated Functional Genomics (IFG), University of Muenster, Roentgenstraß 21, 48149 Muenster, Germany; ^6^KEK GmbH, Kemberger Straße 5, 06905 Bad Schmiedeberg, Germany; ^7^University of Applied Science Jena, Carl-Zeiss-Promenade 2, 07745 Jena, Germany; ^8^Zurich Center for Integrative Human Physiology (ZIHP), University of Zurich, Winterthurerstraß 190, 8057 Zurich, Switzerland; ^9^Study Group “Magdeburger Arbeitsgemeinschaft für Forschung unter Raumfahrt- und Schwerelosigkeitsbedingungen” (MARS), Otto-von-Guericke-University Magdeburg, Universitätsplatz 2, 39106 Magdeburg, Germany

## Abstract

Cells of the immune system are highly sensitive to altered gravity, and the monocyte as well as the macrophage function is proven to be impaired under microgravity conditions. In our study, we investigated the surface expression of ICAM-1 protein and expression of ICAM-1 mRNA in cells of the monocyte/macrophage system in microgravity during clinostat, parabolic flight, sounding rocket, and orbital experiments. In murine BV-2 microglial cells, we detected a downregulation of ICAM-1 expression in clinorotation experiments and a rapid and reversible downregulation in the microgravity phase of parabolic flight experiments. In contrast, ICAM-1 expression increased in macrophage-like differentiated human U937 cells during the microgravity phase of parabolic flights and in long-term microgravity provided by a 2D clinostat or during the orbital SIMBOX/Shenzhou-8 mission. In nondifferentiated U937 cells, no effect of microgravity on ICAM-1 expression could be observed during parabolic flight experiments. We conclude that disturbed immune function in microgravity could be a consequence of ICAM-1 modulation in the monocyte/macrophage system, which in turn could have a strong impact on the interaction with T lymphocytes and cell migration. Thus, ICAM-1 can be considered as a rapid-reacting and sustained gravity-regulated molecule in mammalian cells.

## 1. Introduction

Several limiting factors for human health and performance in microgravity have been clearly identified arising from the immune system, and substantial research activities are required in order to provide the basic information for appropriate integrated risk management. The gravity-sensitive nature of cells of the immune system renders them an ideal biological model in search for general gravity-sensitive mechanisms to understand how the architecture and function of human cells are related to the gravitational force and therefore adapted to life on Earth. Cells of the immune system are highly sensitive to altered gravity (for review see [1–4]). T lymphocytes as well as monocytes and macrophages are impaired severely in their functions under microgravity conditions [2–4]. T cell activation is severely disturbed under microgravity conditions as shown in the blood of astronauts during and after space flight [5] and in numerous* in vitro* experiments (reviewed by [6]). In monocytes the secretion of the cytokines IL-1, IL-6, TNF-alpha, and IL-10 is altered under microgravity conditions [7, 8]. Substantial changes in gene expression of monocytes and in gene induction associated with the differentiation of monocytes into macrophages have been observed [8].

Migration and adhesion of immune competent cells at areas of infection, inflammation, or structural disorders are indispensable for the immune response [9]. For these processes the communication and connection between cells are essential. The integrins of the LeuCAM family (LFA-1 and MAC-1) and their ligands, the intercellular adhesion molecules (ICAMs), are receptors that mediate the attachment between cells (cell-cell contact) and of cells and the extracellular matrix (cell-matrix contact) [10]. ICAMs are transmembrane proteins that are expressed on epithelial cells, endothelial cells, and cells of the immune system including T cells and macrophages. Binding of ICAM-1 (CD54) to receptors on endothelia of blood vessels enables leucocytes to attach and migrate through the endothelia to sites of inflammation [11]. Later on in the immune reaction close and strong interaction between ICAM-1 and LFA-1 is indispensable for the immunological synapse formation between T cells and antigen-presenting cells such as monocytes [12].

ICAM-1 expression is known to be upregulated during mechanical stress [13], in a long-term microgravity environment [14], in the NASA-developed Rotary Cell Culture Systems (RCCS) as well as during short-term microgravity in parabolic flights [15] in endothelial cells. While these studies show gravity sensitivity of ICAM-1 in endothelial cells, less is known about the effects of microgravity on cells of the 2 monocyte/macrophage system (MMS). Therefore in this study we investigate whether the ICAM-1 surface expression is regulated by altered gravity in these cell types. The MMS belongs to the innate immune system and represents the body's first line of defense. The innate immune system is characterized by a fast, but unspecific immune reaction, and it activates the adaptive immune response. This activation occurs through interaction of antigen-presenting cells (APCs)—dendritic cells and macrophages [16]—with T lymphocytes. Macrophages are relatively long-lived, carry a variety of surface receptors, and reside in many tissues including the gastrointestinal tract, the respiratory tract, the liver, the spleen, bones, and connective tissues [17]. Microglial cells are the brain-resident macrophage population which crucially controls and regulates immune reactions inside the central nervous system (CNS).

In our study, we investigated the surface expression of ICAM-1 protein and expression of ICAM-1 mRNA in cells of the monocyte/macrophage system in microgravity. As cell models we used primary cells (macrophages, T cells) as well as cell lines (U937 myelomonocytic cells, macrophage-like differentiated U937 cells, and BV-2 microglial cells). We conducted experiments with different durations of microgravity in clinorotation, parabolic flight, sounding rocket, and orbital flight experiments.

## 2. Methods

### 2.1. U937 Cell Culture and Macrophage-Like Differentiation

U937 cells (ATCC CRL-1593.2) are a human monocytic cell line that preserves the main monoblastic characteristics of monocytes including the ability to differentiate into a macrophage-like phenotype. U937 cells were cultured in RPMI 1640 medium with or without 20 mM HEPES (Biochrom, Berlin, Germany), supplemented with 10% fetal calf serum (FCS, Biochrom) or 10% human serum (HS, Biowest), 2 mM glutamine (PromoCell), 100 U/mL penicillin, and 100 *μ*g/mL streptomycin (Gibco). Subcultivation was done at a cell density of 1 × 10^6^ cells/mL. Stimulation and differentiation were performed by adding 25 nM phorbolmyristylacetate (PMA) (Sigma-Aldrich) in dimethylsulfoxid (DMSO, 0.1%) (Sigma-Aldrich) at a cell density of 0.5 × 10^6^ cells/mL. Differentiation medium was supplemented with 10% HS, 2 mM glutamine, 100 U/mL penicillin, and 100 *μ*g/mL streptomycin. Cells were differentiated on polycarbonate (Makrolon) slides (SIMBOX/Shenzhou-8) or in cell culture flasks (parabolic flights and clinorotation experiments) for 72 h at 37°C with 5% CO_2_ into a macrophage-like phenotype. U937 macrophage-like cells were detached by Macrophage Detachment Solution DXF (PromoCell) following manufacturer's protocol. After detaching, cells were filled immediately into Nutrimix bags (B. Braun Melsungen) (1-2 × 10^7^ cells in 10 mL medium each bag) for parabolic flight or in serological pipettes (1 mL cell suspension with 0.25–0.5 × 10^6^/mL) for clinorotation experiments [18, 19].

### 2.2. Primary Human Macrophages

Human primary M2 macrophages (PromoCell) were cultivated with M2-Macrophage Generation Medium DXF (PromoCell). Cells were detached by Macrophage Detachment Solution DXF (PromoCell) following the manufacturer's protocol. After detaching, cells were filled immediately into Nutrimix bags (B. Braun Melsungen) (2 × 10^6^ cells in 10 mL medium each bag) for parabolic flight, or in serological pipettes (1 mL cell suspension with 0.25–0.5 × 10^6^/mL) for clinorotation experiment [18, 19].

### 2.3. BV-2 Microglia Cell Culture

Since primary microglia are not available in quantities required for parabolic flight experiments, we used the murine cell line BV-2, whose function resembles that of tissue macrophages and which share many properties with both peripheral macrophages and monocytes [20]. BV-2 microglial cells were cultured in DMEM medium (Biochrom, Berlin, Germany) supplemented with 10% FCS and without antibiotics. 72 hours before parabolic flight experiment, the cells were set on only 2% FCS (serum starved) for the transport and were supplemented again with 10% FCS after arrival. Before transport to Bordeaux-Mérignac airport, BV-2 cells were transferred into 200 mL Nutrimix bags (B. Braun Melsungen, Melsungen, Germany) at a density of 3 × 10^6^ cells in 15 mL medium.

### 2.4. Experiments in Simulated Microgravity (2D Clinorotation)

**Figure 1:**
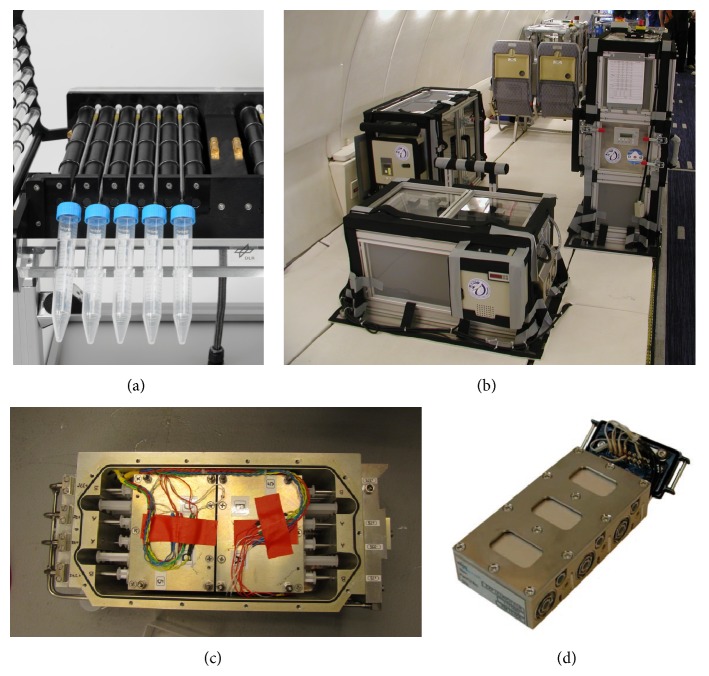
Technology for cell culture experiments in different microgravity research platforms. (a) Fast-rotating two-dimensional (2D) clinostat manufactured by the German Aerospace Center (DLR, Cologne, Germany) was used to provide simulated microgravity. Under the chosen experimental conditions (60 rpm, 4 mm pipette diameter) a maximal residual acceleration of 4 × 10^−3 ^g is achieved at the outer radius of the pipette and decreases towards the center. (b) Experimental hardware structure which consists of an incubator rack to store the cell containers temporarily before the experiment at 37°C (left), an experimental rack in which all active aggregates are accommodated and where the living cells are handled during altered gravity (right) and a cooling rack to temporarily store all cell containers after the injection of the stop/fixation liquid at 4°C until landing (front). (c) Payload of TEXUS-49 sounding rocket tempered and vacuum-resistant container with experiment syringe systems. (d) Plunger unit EUE for SIMBOX (Science in Microgravity Box) incubator system, support structure (housing made of PEEK), which includes three culture chambers and six supply units, two for each culture compartment. Each culture chamber represents an independent loop. The culture chambers filled with medium are closed on the top of the housing by means of polycarbonate specimen window slides, where the adherent cells are attached beforehand. The housing is tightened by silicon sealing and covered by an aluminum plate (cover) fixed with screws.

A fast-rotating two-dimensional (2D) clinostat manufactured by the German Aerospace Center (DLR, Cologne, Germany) was used to provide simulated microgravity (Figure 1(a)). The principle of clinorotation-induced microgravity is the rotation of a cell suspension in a serological pipette perpendicular to the Earth's gravity. The microgravity produced is an averaging of the gravity vector, if the clinostat rotates with 40–100 rpm. Under the chosen experimental conditions (60 rpm, 4 mm pipette diameter) a maximal residual acceleration of 4 × 10^−3 ^g is achieved at the outer radius of the serological pipette and decreases towards the center. The clinostat device was placed in an incubator providing constant 37°C. Fifteen serological pipettes rotated at the same time with 60 rpm. 1 g controls were placed at the ground plate of the clinostat without rotation but the same environmental conditions are as the *μ*g samples. The density of the cell suspension was 0.5 × 10^6^/mL (U937 macrophage-like cells), 0.25–0.5 × 10^6^/mL (human primary macrophages), or 0.75 × 10^6^/mL (BV-2 microglial cells) in 1 mL volume each. The duration of the filling procedure was not longer than 10 min for all 30 serological pipettes. Cells were cultured in the serological pipettes for 24 h–96 h. After clinorotation, cells were fixed by the addition of 500 *μ*L of 3% PFA (Sigma-Aldrich)/2% sucrose (Sigma-Aldrich) solution for 30 min, washed with PBS and analyzed after immunocytochemical staining by flow cytometry.

### 2.5. Parabolic Flights as Microgravity Research Platform

During a parabolic maneuver, an aircraft is weightless by flying on a Keplerian trajectory, described as an unpropelled body in ideally frictionless space subjected to a centrally symmetric gravitational field [21]. During this free-fall trajectory, the resultant of all forces acting on the aircraft other than gravity is zeroed. During a flight campaign, which normally consists of three individual flights, 31 parabolas are flown on each flight, with 93 parabolas in total. On each parabola, there is a period of increased gravity (1.8 g) which lasts for 20 seconds immediately prior to and following the 20-second period of reduced gravity (acceleration in *x*-, *y*-, and *z*-axis was below 2 × 10^−3 ^g at all times during the *μ*g parabola, Figure 1(a)). During the parabolic flight maneuver, the aircraft gradually pulls up its nose and starts climbing at an angle of approximately 45 degrees. This phase lasts for about 20 seconds, during which the aircraft experiences an acceleration of around 1.8 g. The engine thrust is then reduced to the minimum required to compensate for air-drag, and the aircraft is then in a free-fall condition, lasting approximately for 20 seconds, during which weightlessness is achieved. At the end of this phase, the aircraft must pull out the parabolic arc, a maneuver which gives rise to another 20 second period to 1.8 g on the aircraft, after which it returns to normal level flight attitude. Special designated flight areas were above the Atlantic Ocean and the Mediterranean Sea. Experiments were conducted during the 10th and 19th DLR parabolic flight campaigns of the German Aerospace Center (“Deutsches Zentrum für Luft- und Raumfahrt”, DLR) in Bordeaux, France. The campaign used the only large aircraft that is licensed in Europe to perform parabolic flights for research purposes, the Airbus A300 ZERO-G. This aircraft is a specially configured test aircraft operated by NOVESPACE (Bordeaux, France) according to the standing orders of NOVESPACE (A300 ZERO-G Rules and Guidelines RG-2001-1, RG-2008-1, RG-2008-2, RG-2009-1, and RG-2009-02) and the CEV (Centre d'essai en vol).

### 2.6. In-Flight Hardware for Parabolic Flight Experiments

A custom-made hardware meeting the requirements for experiments with human cell culture on board the Airbus A300 ZERO-G was developed in collaboration with KEK GmbH, Germany (Figure 1(b)). The system has already been used successfully for cell culture experiments during 9 parabolic flight campaigns [18, 19]. The system consists of double-sealed cell containers holding the cells of the monocyte-macrophage system and three experimental modules that supply storage of samples before the experiment, half-automated performance of the experiment, and storage of the processed samples. The first module holds the cell containers at 36.5°C in a hanging position. From there, containers are transferred into the second module manually. In this module, cells were fixed by the addition of fixation reagent upon triggering. Triggering was done manually at defined time intervals (20 sec) after the onset of the gravitational condition of interest. The third experimental module served as in-flight storage for the fixated samples at 4°C. Three samples could be processed in parallel. Sample exchange required approximately one minute of a defined procedure by three trained persons.

### 2.7. Procedures during Parabolic Flight Experiments

Transport of in-flight cell culture bags in in-flight-configuration and of fixed samples after the parabolic flight was provided by the Swiss Air Force from Zurich to Bordeaux during each flight day of the 13th DLR Parabolic Flight Campaign or by train during each flight day of the 19th DLR Parabolic Flight Campaign. After arrival at the flight location on the evening before the flight, cells were incubated overnight at 37°C and handled very carefully in order to avoid any mechanical or temperature cell stress. All steps of the entire cell preparation and transport procedure had been tested extensively with respect to cell viability and function beforehand. All procedures during the parabolic flight campaign had been tested several times and highly standardized following an extensive and detailed standard protocol. During the campaign, all procedures were documented and double-checked. In-flight *μ*g and 1 g control experiments were performed in 200 mL Nutrimix bags [18, 19] used as in-flight cell culture bags containing 3 × 10^7^ cells in 15 mL. During the onset of *μ*g or during 1 g (in-flight control experiments), 10 ng PMA/mL (with 0.01 residual DMSO) or 10 ng TNF-*α*/mL or plain cell culture medium were added to the cells. After 20 sec of *μ*g or 1 g, cells were fixed by addition of 1% formaldehyde (Sigma-Aldrich) (for cytometry analysis) or lysed by RLT buffer (Qiagen) (for RNA analysis) and cooled immediately (4°C) during the remaining flight. Experiments were performed at least three times during independent flights and separate flight days. After the flight, fixed cells were transported to the laboratories on the same day, harvested, and subjected to analysis.

### 2.8. TEXUS-49 Sounding Rocket Experiment

For the TEXUS-49 campaign at ESRANGE (Kiruna, Sweden), U937 cells were cultivated in the fully installed laboratories on site. Cells were seeded with a density of 0.2 × 10^6^ cells/mL and the medium was exchanged every 48 hours as described above. On the launch day, cells were visually inspected, harvested, counted, and pooled to a concentration of 5 × 10^7^ cells/mL. 0.5 mL of this cell suspension was filled into a sterile 3 mL plastic syringe shortly before the launch. Additionally, one syringe was filled with 0.3 mL of cell culture medium and another one with 1 mL Trizol LS (Life Technologies, Germany). The three syringes were mounted on a plastic block with a tubing system connecting them. This unit was finally integrated into the automatically operated experiment system (Figure 1(c)). In total 35 of these experiment units were prepared and kept at 37°C until the integration into the payload of the rocket. During the experimental run, first the 0.3 mL of medium, as a potential placeholder for an activation solution, and then the 1 mL of Trizol LS were injected to the cell suspension at defined time points to lyse the cells and preserve the current status of differential gene expression. Injections were performed at 75 sec after launch to monitor a so-called baseline (BL) directly before the *μ*g phase, and at 375 sec after launch at the end of the *μ*g phase. A group of 1 g ground controls was treated immediately after the *μ*g sample group. TEXUS-49 consisted of a VSB-30 engine (S-30 solid rocket stage with an S-31 second stage) and of the payload. The rocket was launched on March 29, 2011, at 06:01 am from the ESRANGE Space Center near Kiruna, Sweden. During the ballistic suborbital flight, an altitude of 268 km and 378 sec of microgravity with a quality of 10^−5 ^g were achieved.

### 2.9. SIMBOX Incubator System with Plunger Experiment Insert

SIMBOX (Astrium GmbH Friedrichshafen, Germany; Kayser Italia, Livorno, Italy) is a programmable, space-qualified incubator for biological research in space equipped with a 1 g in-flight centrifuge for 1 g control experiments. The incubator allows for fully automatic execution of biological experiments with limited use of commands during orbital flight in a controlled thermal environment. The SIMBOX incubator (internal volume 34 liters, dimensions 461 × 551 × 273 mm, empty mass 16 kg, fully integrated mass 34.5 kg, max. power 130 W) accommodates 40 experiment unique equipment (EUEs) with 24 EUEs on the *μ*g-platform and 16 EUEs on the 1 g-centrifuge. The plunger experiment insert (Figure 1(d)) was developed by Astrium GmbH and is described in the* Astrium Space Biology Product Catalog* [22]. It allows medium exchange and chemical fixation of adherent cell cultures. There are two plungers which can be filled with any liquid and automatically activated to inject it into the experimental volume. The EUEs consisted of a support structure (housing made of PEEK) which includes three culture chambers (CCs) and six supply units (SU, plungers), two per culture compartment. Each CC has two SUs and represents an independent loop. The CCs are closed on the top of the housing by Specimen Slides (SS) made of polycarbonate, on which the adherent cells were attached. The chamber (covered by the window slide) contained the medium. The housing is tightened by silicon sealing and covered with an aluminum plate (cover), which is fixed with screws. The container lid of the Biorack standard type I container is mounted onto the housing. The Biorack standard is based on the accommodation of various EUEs into experiment containers which provide the interface to facilities and support infrastructure [22]. The plunger unit is qualified for an unmanned capsule mission and for use on the International Space Station (ISS).

The unmanned Shenzhou-8 spacecraft was launched on October 31, 2011, at 21:58 UTC (November 1, 2011, 05:58 LT) on board of a Long March 2F (CZ-2F) rocket from the Jiuquan Satellite Launch Center (JSLC) in Inner Mongolia. On November 17, the capsule was autonomously deorbited and landed at 12:38 UTC (20:38 LT) around 500 km north of Beijing. The SIMBOX was recovered immediately and transported by helicopter and jet aircraft to the PITC, Beijing. Total early retrieval time was 6 hours. On arrival at the PITC, the SIMBOX was opened, and the EUEs were removed and inspected. The samples were recovered and stored in cold (4°C) PBS until arrival in Zurich for analysis.

### 2.10. SIMBOX Experiment Execution

Medium was changed before integration of the slides into the EUEs. Inside the EUEs, the slides were bedded in 0.5 mL fully CO_2_ saturated RPMI 1640 medium with 10% HS, 2 mM glutamine, 100 U/mL penicillin, 100 *μ*g/mL streptomycin, and 250 ng/mL amphotericin B (PromoCell). Bellow 1, 3, and 5 of the EUEs [23] were filled with RPMI 1640 medium, 10% HS, 100 U/mL penicillin, 100 *μ*g/mL streptomycin, 250 ng/mL amphotericin B, 2 mM glutamine, 1% PFA, and 0.6% sucrose. Bellow 2, 4, and 6 of the EUEs [23] were filled with PBS, 100 U/mL penicillin, 100 *μ*g/mL streptomycin, and 250 ng/mL amphotericin B. Two EUEs (6 chambers) were prepared for the *μ*g-position, and one EUE (3 chambers) was prepared for the 1 g position. The gravity vector of the 1 g position was perpendicular to the surface of sample slides (*z*-axis). During the unpowered transport from the laboratory to the spacecraft and installation in the spacecraft (total time 3 h 06 min), the temperature was always above 21°C. Shenzhou-8 launch was on October 31, 2011, 21:58 UTC and the spacecraft attained orbit at 22:08 UTC. The SIMBOX timeline started at 22:34 UTC. Active temperature control was set to 23°C. The centrifuge speed for the 1 g reference centrifuge was 74.40 rpm. Plungers 1, 3, and 5 of all three EUEs were activated between 120:50:00 and 120:55:20 (hours:min:sec) of the timeline sequences of 40 seconds. Plungers 2, 4, and 6 of all three EUSs were activated between 122:50:00 and 122:55:22 (hours:min:sec) of the timeline sequences of 40 seconds. Human macrophage-like U937 cells were cultivated for 5 days inside the SIMBOX hardware on board of the Shenzhou-8 spacecraft in *μ*g and 1 g conditions, fixed with 1% PFA/sucrose solution for 2 h, and stored in PBS on board at 23°C until landing. After landing, the polycarbonate slides were removed, washed, and then stored in PBS at 4°C for 2 weeks until analysis. The ground control experiment was executed analogously to the flight scenario. Details about the experiment were published previously [23].

### 2.11. Quantification of ICAM-1 by Flow Cytometry

Surface expression of ICAM-1 on BV-2 microglial cells and U937 monocytic and macrophage-like cells as well as primary human macrophages was analyzed by flow cytometry. Cells were collected from the Nutrimix bags (parabolic flight) or standardized serological pipettes (clinorotation) fixated in PFA/sucrose solution. After the washing procedure (PBS without Ca/Mg, Biochrom) cells were stained with ICAM-1 monoclonal antibody (BV2: Invitrogen, FITC labeled; U937 and primary macrophages: cell signaling, PE labeled). Analysis was performed using a flow cytometer (FACSCanto II, BD Biosciences, Heidelberg, Germany), collecting at least 20000 cells per sample. Mean fluorescence intensity Ratio (MFI) was calculated as MFI of sample/MFI of isotype control.

### 2.12. ICAM-1 Analysis in BV-2 Cells from Parabolic Flight Experiments

Cells were quadruple stained for ICAM-1, apoptosis (TUNEL), cell delineation (HCS cell mask), and DNA (DAPI). In brief, cells were cytospinned onto glass slides washed 3x with PBS, permeabilized for 1 min with 0.1% Triton-X 100 (Sigma-Aldrich, Buchs, Switzerland), and washed again 3x with PBS and incubated with the TUNEL labeling mix (Boehringer, Mannheim, Germany) according to the manufacturer's instructions. For TUNEL staining, rhodamine coupled dUTP was used. Subsequently to overnight incubation, cells were washed again 3x with PBS, blocked with 0.5% BSA, and stained with FITC labeled ICAM-1 antibody (BD Pharmingen, San Jose, USA) at a concentration of 0.05 mg/mL for 2 h. After additional washing, cells were stained entirely with HCS cell mask deep red cytoplasmic and nuclear stain (Invitrogen, Basel, Switzerland) using a dilution of 1 : 20000 and nuclei were labeled with DAPI (Invitrogen) at 1 *μ*g/mL for 10 min. Labeled cells were imaged using a Leica microscope DMI 6000 and LAS AF software (Leica Microsystems, Wetzlar, Germany). For automated imaging, the unified random sampling module was utilized, 63 randomized images of each sample were recorded, and at least 500 single cells from 3 independent experiments from 3 different parabolas were analyzed. From each image cells were identified according to the following criteria: nucleus of a predefined size and brightness, being TUNEL negative, and containing HCS staining over a certain threshold. Surface calculation of these cells was performed with Imaris and automated for all images using batch coordinator (Bitplane AG, Zurich, Switzerland). Therefore, the mean intensity of the ICAM-1 signal was analyzed in living cells exclusively and binned into ICAM-1 intensity categories of 50 gray levels. Statistical analysis was carried out using GraphPad Prism software (GraphPad Software, Inc., La Jolla, USA) and Student's *t*-test was applied for all analyzed data.

### 2.13. ICAM-1 Analysis in Differentiated U937 Cells from the SIMBOX Experiment

Polycarbonate slides were cut by a water jet method into 16 T-shaped pieces. Each piece was stained individually. In order to differentiate between dead (necrotic/apoptotic) and living cells before fixation, slides were stained with CellMask-deep red plasma membrane stain (Invitrogen) and TUNEL reagent (Fluorescein-12-dUTP, Roche). In addition, cells were labeled with different mono- and polyclonal primary antibodies directed against the cytoskeleton components and immunological relevant surface molecules (reported in [17]) and ICAM-1 in concentrations according to the manufacturers' protocols. After blocking with 1% BSA in PBS for 1 h, primary antibodies were detected by species specific secondary antibodies used in a dilution of 1 : 1000 in 0.5% BSA in PBS. Secondary antibodies were labeled with Alexa-Fluor405 or Alexa-Fluor568 (Invitrogen). Slide pieces were analyzed by confocal laser scanning microscopy (Leica, SP5). Only cells positive for CellMask and negative for TUNEL were subjected to further analysis, since these represent the living cell population in the experiment. Digital image analysis was performed using Imaris software (Bitplane).

### 2.14. RNA Isolation from the Parabolic Flight Experiments

After the return of the aircraft and transport of the samples to the on-site laboratory facilities, the containers were disassembled, the Nutrimix bags were gently agitated, and the lysed cell solution from each bag was filled into a T75 straight neck cell culture flask. The cell solution was vortexed for 10 sec and passed four times through a *Ø* 0.8 × 120 mm needle (B. Braun Melsungen, Germany) fitted to a 50 mL syringe. 50 mL of absolute ethanol was added and precipitates were resuspended by vigorous shaking. A valve and a sterile connective piece were placed on a Qiavac 24 plus vacuum system (Qiagen, Germany) and an RNA maxi column (Qiagen, Germany) was attached to the connective piece. A vacuum of −200 mbar was adjusted and the column was loaded with the lysed cell suspension. Then, the valve was closed and the column was centrifuged at 4000 g for 3 min. 15 mL of buffer RW1 (Qiagen, Germany) was applied for washing membrane bound RNA. After centrifugation at 4000 g for 7 min, the flow was discarded, and two washing steps with 10 mL RPE buffer (Qiagen, Germany) followed each with centrifugation at 4000 g for 3 min and 10 min, respectively. The column bound RNA was eluted by application of 600 *μ*L of RNase-free water (Qiagen, Germany), incubation for 1 min at room temperature, and centrifugation for 4 min at 4000 g. The elution step was repeated with the first eluate. The RNA was transported at approximately −150°C in a Cryo Express dry shipper (CX-100; Taylor-Wharton, USA) prepared with liquid nitrogen and stored at −80°C until the processing of the RNA for the microarray analysis.

### 2.15. RNA Isolation during the TEXUS-49 Sounding Rocket Campaign

Directly after landing, localization, and recovery of the payload, the experiment modules were dismantled and handed over to the scientists. The cell suspension was sheared three times with a 20 G needle (B. Braun Melsungen, Germany) and distributed in two 2.0 mL tubes. 0.1 mL of chloroform (Sigma-Aldrich, Germany) was added, and the solution was vortexed for 15 sec and incubated for 5 min at room temperature before a 15 min centrifugation step at 11000 g and 4°C. The upper phase of both 2.0 mL tubes was transferred into a 15 mL tube and 4 mL of RLT buffer and 3 mL of absolute ethanol were added and mixed. 4 mL of this solution was pipetted on an RNA Midi column (Qiagen, Germany) and centrifuged for 30 sec at 3000 g at room temperature. The flow was discarded, and the residual 4 mL of RNA solution was loaded on the column and centrifuged for 5 min at 3000 g at room temperature. Then, the columns were washed twice with 2.5 mL of RPE buffer and centrifuged for 2 min and 5 min, respectively, at 3000 g and room temperature. The RNA was eluted by the addition of 250 *μ*L RNase free water (Qiagen, Germany) to the column, incubation for 1 min at room temperature, and centrifugation for 3 min at 3000 g and room temperature. The eluate was loaded again onto the column, followed by a 1 min incubation and centrifugation for 5 min at 3000 g, and room temperature. The isolated RNA was transferred into sterile Cryo-tubes and stored until the return transport at approximately −150°C in a Cryo Express dry shipper (CX-100; Taylor-Wharton, USA) prepared with liquid nitrogen. After arrival in the home laboratory, samples were stored at −80°C until processing the RNA for the microarray analysis.

### 2.16. RNA Processing and Microarray Analysis for Parabolic Flight and TEXUS-49 Sounding Rocket Campaign Samples

RNA quantity and purity were analyzed spectrophotometrically using a Nanodrop 1000 (Thermo Scientific). Isolated RNA samples were all of high quality with 260/280 nm ratios between 1.9 and 2.1. The RNA integrity was measured using an Agilent 2100 Bioanalyzer (Agilent Technologies, USA). Only RNA with an RNA Integrity Number (RIN) > 8.7 was used for the following microarray analysis. 400 ng total RNA was applied to Cy3-labeling with the “Low RNA Input Linear Amplification Kit, PLUS, One-Color” (Agilent Tech-nologies) and hybridized for 17.5 h to a NimbleGen expression microarray (12 × 135000 features) employing the “Gene Expression Hybridization Kit” (Agilent Technologies, USA). Afterwards, arrays were washed and scanned by the Micro Array Scanner G2505B (Agilent Technologies, USA).

The image files of the scanner were analyzed with the NimbleScan Software 2.6 using the Robust Multi-Array Analysis (RMA) with the default parameters. RMA, a probe-level summarization method, identifies probes that are outliers in the overall behavior of the expression measured for a given gene. The contribution of outlier probes is reduced in the reported gene expression level, which has been demonstrated to improve the sensitivity and reproducibility of microarray results. In addition to screening outlier probes, NimbleScan software's implementation of RMA used quantile normalization and background correction. The normalized microarray data were analyzed using Partek Genomics Suite 6.6. Statistical analysis was performed using the one-way ANOVA and the false discovery rate (FDR) for multiple testing corrections. Further, the coefficient of variation (CV) expressed in percent was calculated, also known as “relative variability,” which equals the standard deviation divided by the mean. Genes of interest were identified and the log2 values of the measured fluorescent intensities returned by the Partek software were back calculated to linear values. Then, means of all values of the same gene generated by different probes were calculated, if at least three values existed excluding outliers. Subsequently, standard deviations were calculated for the means and an unpaired *t*-test with Welch correction was performed to test statistical significance.

### 2.17. Pathway Enrichment Analysis

The pathway enrichment analysis was performed using Partek Genomics Suite 6.6 and the KEGG human pathway library [24, 25]. The *P* values were calculated by the Fisher exact test. Enrichment analysis was applied on the genes showing differential expression with *P* values of <0.05 and fold change >+1.5 or <−1.5.

### 2.18. Statistical Analysis

Data are expressed as median or as median ± SE. Groups contain the analysis of 200–1000 cells (SIMBOX, shown in box-plots) or data of three independent experiments with 1–5 samples (*n* = 3–15, shown in columns). Data were analyzed by one-way ANOVA followed by Wilcoxon or unpaired *t*-test using GraphPad Prism 5. ^*^ *P* < 0.1 was considered to be significant, ^**^ *P* < 0.05 as significant, and ^***^ *P* < 0.01 as very significant.

## 3. Results

### 3.1. Clinorotation of Downregulated ICAM-1 Expression in BV-2 Microglial Cells

**Figure 2:**
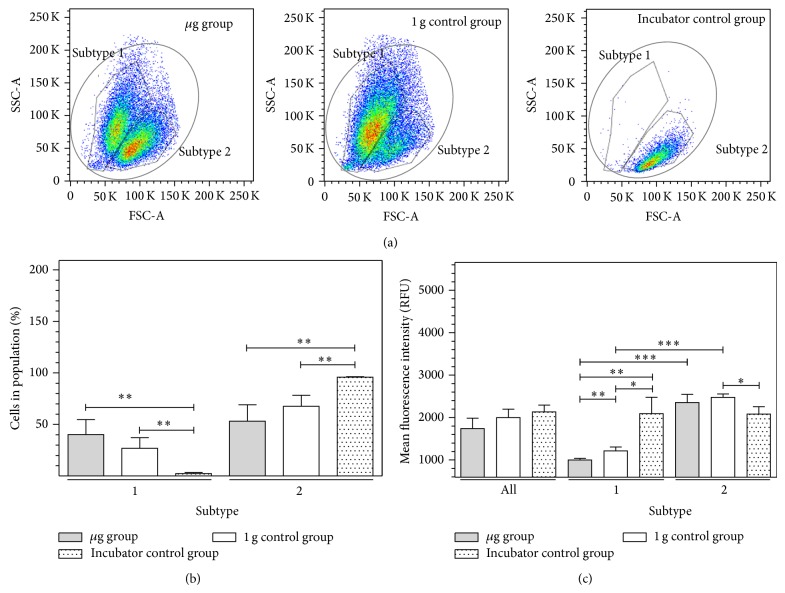
Cytometry analysis of ICAM-1 expression in BV-2 microglial cells in simulated microgravity (2D clinorotation). BV-2 microglial cells were exposed to either clinorotation (*μ*g), placed in the clinostat but not rotated (1 g control group), or cultured under standard cell culture conditions (incubator control) for 24 h. Cells were stained for ICAM-1 surface expression and analyzed by flow cytometry. The level of ICAM-1 surface expression is represented by the mean fluorescent intensity assessed by flow cytometry. (a) In forward/sideward scatter detection mode of flow cytometry, two gates were set to separate two subtypes of BV-2 microglial cells that appeared different in size and granulation (subtypes 1 and 2 in dot plots). (b) Distribution of BV-2 microglial cells in subtypes 1 and 2 after exposure to different gravity conditions. (c) Quantification of ICAM-1 expression after exposure to different gravity conditions within subtypes 1 and 2. Data are given as median ± SE (^*^ *P* < 0.1, ^**^ *P* < 0.05, ^***^ *P* < 0.01, *n* = 3, according to one-way ANOVA followed by Wilcoxon or unpaired *t*-test).

First, we analyzed ICAM-1 expression in BV-2 microglial cells after 24 h clinorotation (60 rpm, 4 mm pipette diameter, maximal residual acceleration of 4 × 10^−3 ^g at the outer radius of the pipette). The clinostat device was placed in an incubator, which provides constant 37°C. Fifteen serological pipettes rotated at the same time with 60 rpm. 1 g controls were placed at the ground plate of the clinostat without rotation but with the same environment condition like *μ*g samples. A 1 g control group of BV-2 cells was filled into 1 mL serological pipettes in the same way as the clinorotation cell group but was not clinorotated. Another control group was kept at regular cell culture conditions in the incubator (37°C, 5% CO_2_). Cells were subsequently stained for cell surface ICAM-1, apoptosis (TUNEL), cell delineation (HCS CellMask), and DNA (DAPI) (Figure 2). ICAM-1 expression analysis by flow cytometry revealed two distinct subtypes of cells in the clinorotated group (*μ*g group) compared to the 1 g control group and the incubator control group consisting of only one subtype, respectively (Figure 2(a)). The first of the two subtypes was small and stronger granulated (subtype 1) than the second subtype, which appears taller but less granulated (subtype 2). Apoptotic cells were excluded from the analysis by TUNEL staining. Subtype 1 could possibly represent an activated state. Subtype 1 was found in the *μ*g group as well as in the 1 g control group, whereas the incubator control did virtually not contain this subtype. Subtype 2 was represented in all three cell groups, *μ*g, 1 g control, and incubator control cell group. However, it was primarily present in the *μ*g and in the incubator control group and less present in the 1 g control group. The population distribution within cell groups is illustrated in Figure 2(b), showing the relative cell numbers of each population in each cell group. Since the incubator control group consisted almost exclusively of cells in subtype 2, this number was nearly 100%, whereas subtype 1 was close to 0%. The *μ*g group had almost as many cells in subtype 2 as in subtype 1 with a slight predominance in subtype 2. In Figure 2(c), the mean fluorescence intensity of the cell subtypes in the different cell groups was depicted. While the ICAM-1 expression in the incubator control group was stable in both subtypes (2158 ± 234.4 RFU versus 2082 ± 171 RFU), and the *μ*g cell group displayed significantly less expression of ICAM-1 in subtype 1 compared to subtype 2. ICAM-1 expression was significantly reduced in the *μ*g group compared to the 1 g control and the incubator control group. Cells in the 1 g control group exhibited a similar ICAM-1 expression distribution as cells from the *μ*g group. The mean fluorescence intensities between subtype 2 of different groups did not change dramatically, except a significant difference between the 1 g control group and the incubator control group. In summary, we suppose that ICAM-1 expression was downregulated in microglia cells in simulated microgravity.

### 3.2. Rapid and Reversible Downregulation of ICAM-1 Surface Expression in BV-2 Microglial Cells in Real Microgravity

In the next step, we investigated the cell surface expression of ICAM-1 in real microgravity provided by parabolic flights in murine BV-2 microglial cells. During parabolic flight experiments, cells were activated at the onset of *μ*g (or during 1 g for in-flight control experiments) by the addition of PMA or TNF-*α* or not activated by the addition of medium only. After a 20 sec period of altered gravity, cells were fixed by the addition of formaldehyde.

During the 13th DLR parabolic flight campaign, we also addressed the issue that, during parabolic flight experiments, cells are generally subjected to irregular stress by cell preparation and handling and by the in-flight situation itself. This combination of interference factors always leads to a significant degree of damaged or dead cells, which could affect the experiment results and mask a possible microgravity-related effect, even under presence of internal controls. For this reason, we developed an automated analysis method, which allows for the specific analysis of alive and morphologically intact cells at the moment of fixation.

**Figure 3:**
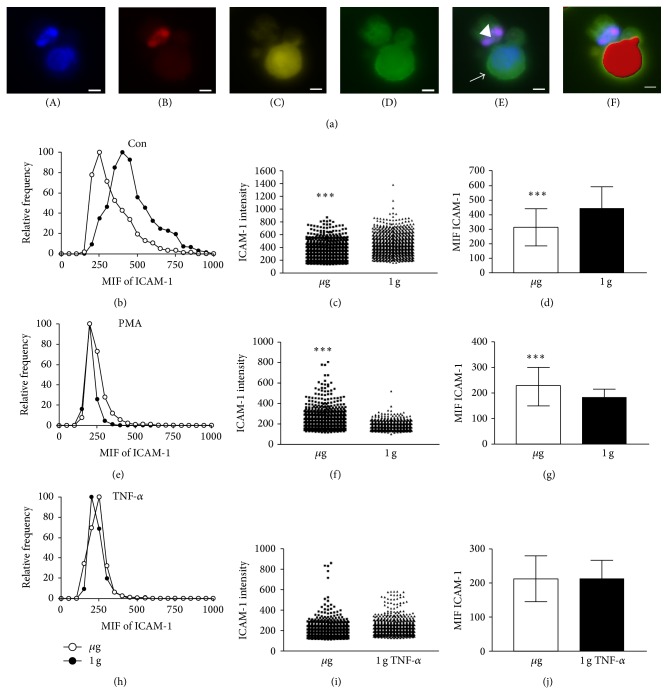
ICAM-1 surface expression reacts rapidly and reversibly to microgravity. (a) Microscopy of ICAM-1, TUNEL, HCS CellMask, and DAPI including surface calculation for HCS. In order to identify nuclei, cells were stained with DAPI (A). Apoptotic cells were identified by TUNEL reaction (B) and HCS CellMask label (C) which can be retained to a higher extend in nonapoptotic cells. ICAM-1 intensity is depicted in (D). A merge of TUNEL, DAPI, and ICAM-1 (E) shows an apoptotic cell (◄) and a living cell (→). The automated calculation of an iso-surface is exclusively done for living cells using the HCS CellMask channel as shown in the merge with TUNEL, DAPI, and ICAM-1 (F). (b)–(j) BV-2 microglial cells were treated with PMA ((e), (f), and (g)) or TNF-*α* ((h), (i), and (j)) at the onset of microgravity or during the 1 g in-flight control phase or left untreated ((b), (c), and (d)). Cells were fixed in flight after 20 sec normogravity (1 g) (-●-) or 20 sec microgravity (*μ*g) (-○-). Cells were stained, imaged, and analyzed as described above. The mean intensity of the ICAM-1 signal was binned into mean intensity fluorescence (MIF) categories and the number of cells (frequency) is plotted against these intensity categories ((b), (e), and (h)). ICAM-1 fluorescence intensity of all analyzed cells ((c), (f), and (i)) is depicted for normogravity (triangles) and microgravity (squares). Mean ICAM-1 fluorescence intensity of all analyzed cells ((d), (g), and (j)) was pooled for normogravity (black bar) and microgravity (open bar). For automated imaging, the unified random sampling module was utilized and 63 randomized images of each sample were recorded and at least 500 single cells from 3 independent experiments from 3 different parabolas were analyzed. Mean intensity and SEM are shown and student's *t*-test showed highly significant difference of the fluorescence values of  ^***^ *P* < 0.0001, *n* = 3.

Experiments from different parabolas (1 g and *μ*g, resp.) and different flights were analyzed. The experiments were performed in a sequence of three consecutive *μ*g and 1 g phases. A quadruple fluorescent staining was performed using TUNEL (rhodamine) for detection of apoptotic cells, DAPI for the nuclei, high content screening (HCS) CellMask deep red for the delineation of cells, and FITC-labeled anti ICAM-1 antibody for identification of cell surface expression of ICAM-1. Cells were imaged with a widefield microscope (Leica Microsystems, Wetzlar, Germany) using the uniform random sampling module and identified by an iso-surface calculation (Imaris, Bitplane AG, Zurich, Switzerland). This quadruple staining allowed the exclusion of apoptotic cells in a highly reliable fashion. An example of an apoptotic cell and a living cell is depicted in Figure 3(a). The mean intensity of the ICAM-1 signal was analyzed in nondamaged and nonapoptotic cells only and binned into intensity categories. The relative frequency of these cells was plotted against the fluorescence intensity (Figures 3(b), 3(e), and 3(h)).

We found a rapid and reversible downregulation of ICAM-1 on the surface of BV-2 microglial cells after 20 sec of microgravity, apparent by the frequency of cells expressing ICAM-1 in various intensities (Figures 3(b) and 3(c)) and the mean of ICAM-1 expression intensity (Figure 3(d)) being only 70% in microgravity compared to normogravity. In the presence of PMA, ICAM-1 expression was upregulated (Figures 3(e), 3(f), and 3(g)), whereas the presence of the proinflammatory cytokine TNF-*α* abrogated the microgravity-induced ICAM-1 downregulation (Figures 3(h), 3(i), and 3(j)). Statistical analysis of all pooled data revealed downregulation of ICAM-1 expression in unstimulated microglia upon microgravity to be highly significantly different with *P* < 0.001 (Figure 3(d)). Changes of ICAM-1 expression in PMA stimulated cells were as well highly significant (Figure 3(g)), whereas TNF-*α* stimulation slightly ameliorated the gravity-dependent changes in ICAM-1 expression (Figure 3(j)). Thus, we found a rapid and reversible disappearance of ICAM-1 protein from the cell surface in microgravity.

### 3.3. Increase of ICAM-1 Expression in U937 Human Macrophage-Like Cells and Human Primary Macrophages in Simulated Microgravity

**Figure 4:**
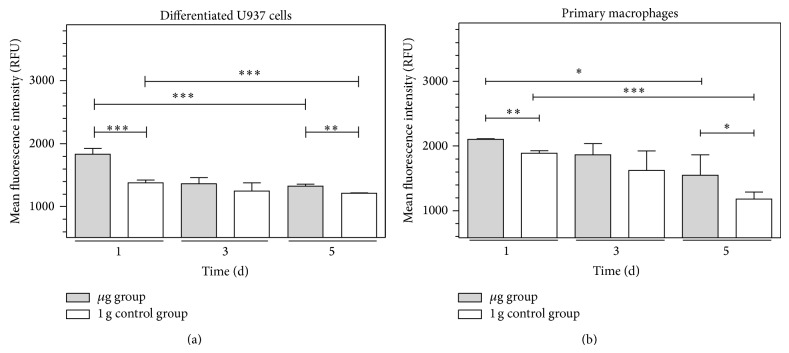
Cytometry analysis of ICAM-1 expression in macrophage-like differentiated U937 cells or primary human macrophages in simulated microgravity (2D clinorotation). Macrophage-like differentiated U937 cells (a) or primary human macrophages (b) were exposed to either clinorotation (*μ*g), placed in the clinostat but not rotated (1 g control group), or cultured under standard cell culture conditions (incubator control). Cells were stained for ICAM-1 surface expression and analyzed by flow cytometry. The level of ICAM-1 surface expression is represented by the mean fluorescent intensity assessed by flow cytometry. (a) Quantification of ICAM-1 expression in macrophage-like differentiated U937 cells after exposure to different gravity conditions for 1 h, 3 h, or 5 h, *n* = 6. (b) Quantification of ICAM-1 expression in primary human macrophages after exposure to different gravity conditions for 1 h, 3 h, or 5 h, *n* = 3. Data are given as median ± SE (^*^ *P* < 0.1, ^**^ *P* < 0.05, ^***^ *P* < 0.01, according to one-way ANOVA followed by Wilcoxon or unpaired *t*-test).

To corroborate the relevance of the results obtained with murine BV-2 microglial cells, we investigated a human macrophage-like cell system. Therefore, human monocytic U937 cells were differentiated into macrophage-like cells [23] and human M2 macrophages were differentiated from blood mononuclear cells. Before the experiment, differentiated macrophage-like cells were detached, resuspended in fresh medium, and filled into 1 mL standardized serological pipettes for the clinostat. Clinorotation was performed for 1 d, 3 d, and 5 d. The 1 g control group of differentiated U937 cells was filled into 1 mL serological pipettes in the same way as the clinorotation cell group but was not rotated. Cells were subsequently fixed and stained for cell surface ICAM-1 and apoptosis (TUNEL) to exclude apoptotic cells from the analysis and subjected to flow cytometry. We detected a highly significant increase of ICAM-1 expression in the clinorotated cells (*μ*g group) compared to the nonrotated cells (1 g control group) after 1 d and 5 d in differentiated U937 cells (Figure 4(a)) and primary macrophages (Figure 4(b)). However, this increase receded after 3 and 5 days of clinorotation. Therefore, we conclude that ICAM-1 expression is increased in human macrophages after 1 and 5 days of simulated microgravity.

### 3.4. Increased ICAM-1 Expression in Differentiated U937 Cells in Real Microgravity during Parabolic Flight

During parabolic flight experiments, we investigated rapid effects of real microgravity on nondifferentiated and differentiated U937 cells and on primary human M2-differentiated macrophages. Nondifferentiated and differentiated myelomonocytic U937 cells were cultured and seeded into Nutrimix bags as described. During the parabolic maneuvers, cells were activated at the onset of *μ*g or during 1 g for in-flight control experiments by the addition of PMA with medium in the case of nondifferentiated U937 cells, or not activated by the addition of medium only in the case of differentiated U937 cells and primary macrophages. After 20 sec microgravity, cells were fixed by the addition of paraformaldehyde. A group of ground control cells was left in Nutrimix bags in the laboratory incubator and activated and fixed after landing in the same experimental equipment. Experiments from different parabolas (1 g and *μ*g, resp.) and different flights were analyzed. A quadruple fluorescent staining was performed using TUNEL (rhodamine) for detection of apoptotic cells, DAPI for the nuclei, high content screening (HCS) CellMask deep red for the delineation of cells, and FITC-labeled anti ICAM-1 antibody for identification of cell surface expression of ICAM-1.

**Figure 5:**
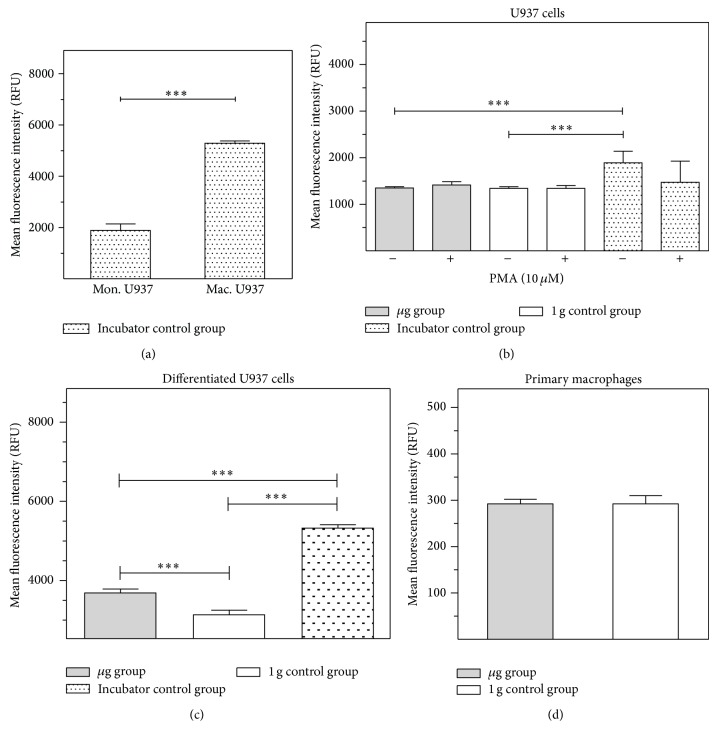
ICAM-1 expression in U937 cells, macrophage-like differentiated U937 cells, and primary human macrophages in different gravity conditions during parabolic flight experiment. ICAM-1 expression was assessed by flow cytometry and fluorescent microscopy following immunocytochemical staining. Cells were cultured under standard cell culture conditions (incubator control) or exposed to different gravity conditions during the 19th DLR parabolic flight campaign. U937 cells were fixed either after PMA-activation in microgravity (*μ*g group) or in 1 g (1 g control group). Differentiated U937 and primary macrophages were fixed after the microgravity phases (*μ*g group) or after the 1 g phases before and after the *μ*g phase (1 g control group). The level of ICAM-1 surface expression is represented by the mean fluorescent intensity assessed by flow cytometry. (a) ICAM-1 surface expression in myelomonocytic U937 cells (mon. U937) and macrophage-like differentiated U937 cells (max. U937) under standard cell culture conditions. (b) ICAM-1 surface expression in U937 cells with and without activation by PMA in different gravity conditions. (c) ICAM-1 surface expression in macrophage-like differentiated U937 cells in different gravity conditions. (d) ICAM-1 surface expression in primary macrophages in different gravity conditions. Data are given as median ± SE (^*^ *P* < 0.1, ^**^ *P* < 0.05, and ^***^ *P* < 0.01, according to one-way ANOVA followed by Wilcoxon or unpaired *t*-test).

*Nondifferentiated U937 Cells*. Differentiation of U937 monocytic cells into macrophage-like cells significantly increased the cell surface expression of ICAM-1 (Figure 5(a)). Nondifferentiated U937 did not demonstrate differential expression of ICAM-1 in microgravity: neither in PMA-stimulated myelomonocytic U937 cells, nor in non-stimulated cells, any significant alteration of ICAM-1 expression could be detected in comparison between microgravity and 1 g conditions (Figure 5(b)). The only significant difference could be observed in nonstimulated U937 cells between the ground control group, the *μ*g group, and the 1 g control group. Differences between 1 g ground and 1 g in-flight controls can be attributed to the flight itself (e.g., vibrations, handling of cell containers) and not to an altered gravity.

*Differentiated U937 Cells*. In contrast to nondifferentiated U937 cells, macrophage-like U937 cells displayed a highly significant gravity-dependent change in ICAM-1 expression (Figure 5(c)). In flight, cell surface ICAM-1 was reduced drastically compared to the ground control. In the microgravity group, ICAM-1 expression was enhanced. This finding is consistent with our experiments in simulated microgravity. We suppose that ICAM-1 is upregulated in differentiated macrophage-like cells in microgravity.

*Primary Macrophages*. For the analysis of primary human M2 macrophages, double fluorescence staining was performed using TUNEL (rhodamine) for detection of apoptotic cells and FITC-labeled anti ICAM-1 antibody for identification of cell surface expression of ICAM-1. Unfortunately, the 1 g incubator control was lost during the experiment procedures. Between the 1 g in-flight control and the microgravity group, no differences in ICAM-1 surface expression could be detected in primary human macrophages. However, due to the technical problems and low detected expression levels compared to primary macrophages in clinostat experiments (see Figure 4(b)), the informative value of these results may be limited and it is planned to repeat the parabolic flight experiment with primary human macrophages.

### 3.5. Increased ICAM-1 Expression in Differentiated U937 Cells during Long-Term Microgravity in the SIMBOX Experiment

**Figure 6:**
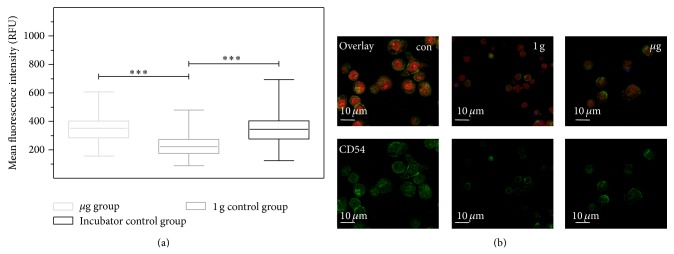
ICAM-1 expression in macrophage-like differentiated U937 cells after long-term exposure to microgravity during the SIMBOX/Shenzhou-8 mission. Cells were cultured under standard cell culture conditions (incubator control) or exposed to different gravity conditions during the SIMBOX/Shenzhou-8 mission. Differentiated U937 cells were fixed in microgravity (*μ*g group) or in 1 g (1 g control group) after 5 days. Only CellMask-positive and TUNEL-negative cells were analyzed. (a) Each group represents analysis of the mean fluorescence of 200–1000 individual cells from one recovered slide. Data are expressed as the median of mean single cell fluorescence intensities with the smallest observation (sample minimum), lower quartile, median, upper quartile, and largest observation (sample maximum). Statistical analysis was performed with GraphPad Prism 5, Wilcoxon test, ^*^ *P* < 0.05, ^**^ *P* < 0.01, and ^***^ *P* < 0.001. (b) Standard cell culture control (con), 1 g hardware control (1 g) and the microgravity sample (*μ*g).

During the SIMBOX (Science in Microgravity Box) mission on Shenzhou-8, we investigated microgravity-associated long-term alterations in macrophage-like differentiated U937 cells and analyzed the effect of long-term microgravity on the cytoskeleton and immunologically relevant surface molecules [23]. Human U937 cells were differentiated into a macrophage-like phenotype and exposed to microgravity or 1 g on a reference centrifuge on orbit for 5 days. The unmanned Shenzhou-8 spacecraft was launched with a Long March 2F (CZ-2F) rocket from the Jiuquan Satellite Launch Center (JSLC) and landed after a 17-day mission. After on-orbit fixation, the samples were analyzed with immunocytochemical staining and confocal microscopy after landing. Double fluorescent staining was performed using HCS CellMask deep red for the delineation of cells and FITC-labeled anti ICAM-1 antibody for identification of cell surface expression of ICAM-1. Cells were analyzed as described above. We detected a significant higher expression of ICAM-1 in long-term microgravity in comparison to the in-flight 1 g control group (Figure 6). Similar to the parabolic flight experiments, incubation of the macrophage-like differentiated U937 cells in the experiment hardware caused a significant downregulation of ICAM-1 expression. Thus, it can be excluded that the microgravity effects on ICAM-1 were caused by the experiment system itself.

### 3.6. No Influence of Altered Gravity on ICAM-1 mRNA

**Figure 7:**
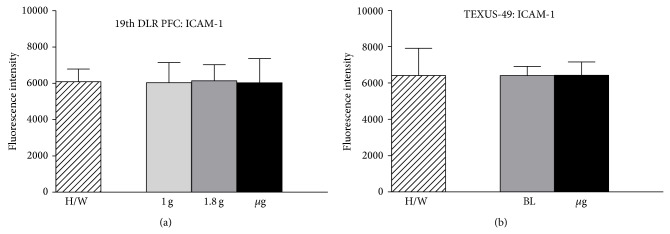
Influence of altered gravity during parabolic flight and sounding rocket flight on ICAM-1 mRNA expression levels. (a) ICAM-1 mRNA expression levels are demonstrated for samples of the 19th DLR parabolic flight campaign after 1 g (light gray), 1.8 g (dark gray), *μ*g (black), and hardware ground controls (H/W, striped) exposure and (b) for samples of the TEXUS-49 campaign after launch and acceleration (BL, dark gray), *μ*g (black), and hardware ground controls (H/W, striped). ICAM-1 fluorescence intensities do not show any significant differences for all compared conditions in both experimental setups. The number of analyzed arrays: 19th DLR PFC: 1 g (*n* = 8), 1.8 g (*n* = 6), *μ*g (*n* = 8) and H/W (*n* = 6); TEXUS-49: H/W (*n* = 6), *μ*g (*n* = 7), BL (*n* = 5).

RNA samples were analyzed for their quantity and quality and further processed for the microarray hybridization on 12 × 135 K Roche NimbleGen arrays. Data from 46 single microarrays (19th DLR PFC: 8x *μ*g, 6x H/W, 8x 1 g, and 6x 1.8 g; TEXUS-49: 7x *μ*g, 6x H/W, and 5x BL) were collected, normalized, and further analyzed. The data tables were screened for ICAM-1 values and mean fluorescence intensities including standard deviations were calculated for all samples of one condition. ICAM-1 shows stable expression for all gravity conditions during the 19th DLR PFC and the TEXUS-49 campaign, as well as for the H/W controls (Figure 7), indicating that microgravity and hypergravity conditions did not have an influence on mRNA ICAM-1 level in the range of 20 seconds until 6 minutes.

### 3.7. Pathway Analysis Reveals an Influence of Real Microgravity on the Natural Killer Cell Mediated Cytotoxicity of Monocytic U937 Cells

Due to the wealth of data microarray analysis provides, we were able to perform a GeneSet enrichment analysis to identify any affected pathways or biological networks in connection with ICAM-1 (see Supplement  1 in the Supplementary Material available online at http://dx.doi.org/10.1155/2015/538786). For the experiments performed during the 19th DLR PFC with monocytic U937 cells, we identified one significantly influenced ICAM-1 related pathway during 20 sec of microgravity compared to the in-flight 1 g control, namely, the natural killer cell mediated cytotoxicity (enrichment *P* value 0.0203328). For the experiments performed on TEXUS-49 during 6 min of microgravity with monocytic U937 cells, we found two weakly altered pathways. Specifically, the NF-kappa B signaling pathway (enrichment *P* value 0.0632651) and the Epstein-Barr virus infection (enrichment *P* value 0.0641782) appeared sensitive to microgravity compared to baseline.

## 4. Discussion

In our study, we investigated the surface expression of ICAM-1 protein and expression of ICAM-1 mRNA in cells of the monocyte/macrophage system in microgravity during clinostat, parabolic flight, sounding rocket, and orbital experiments. In murine BV-2 microglial cells, we found a downregulation of ICAM-1 expression in clinorotation experiments and a rapid and reversible downregulation in the microgravity phase of parabolic flight experiments. In contrast, ICAM-1 expression increased in macrophage-like differentiated human U937 cells during the microgravity phase of parabolic flights and in long-term microgravity provided by a 2D clinostat or during the orbital SIMBOX/Shenzhou-8 mission.

In nondifferentiated U937 cells, no effect of microgravity on ICAM-1 expression could be observed during parabolic flight experiments. A summarizing table, which presents an overview about the cell types tested, the platforms used, the experiment durations, analysis method, the number of experiments, and the detected effects on ICAM-1, is demonstrated in Table 1. In our study and according to previous investigations [26], we detected effects of the experimental hardware, which were controlled by the appropriate hardware control experiments.

In clinostat experiments, subtype of BV-2 microglial cells appeared in the FACS analysis of all clinostat samples (*μ*g or 1 g controls), but not at all in the incubator controls. This subtype consisted of smaller cells and we suppose an “activated” phenotype of BV-2 microglial cells. It is well established that microglial form and function are linked and that cells can cycle reversibly from a simple rounded (activated and amoeboid) to a complex branched form (ramified and resting) [27]. Thus, we assume that microgravity activates microglias cells but downregulates ICAM-1 expression (Figure 2). Control experiments revealed no influence of the serological pipette incubation system on the ICAM-1 expression compared to “normal” cell culture conditions between 1 and 5 d (data not shown).

In this study, we also developed a method that allowed a randomized screening of only those cells that were alive at the fixation time point after the parabola (Figure 3). This is of particular importance because of the damage caused to all cells subjected to a flight experiment. Until now, a method for analyzing only viable and nondamaged cells obtained from flight experiments was lacking. Due to the very limited number of samples during the experiment with BV-2 cells during the 13th DLR PFC, FACS analysis could not be utilized. We therefore developed a microscopy based method to analyze exclusively the living cell portion. Samples were imaged using the uniform random sampling module of Leica LAS AF software in order to fulfill all statistically necessary criteria of randomized sampling. Surface calculation of cells negative for TUNEL label under a certain threshold and positive for HCS CellMask allowed the exclusion of all apoptotic cells. The mean ICAM-1 intensity value of each analyzed cell was taken into account.

Modulation of the expression of surface adhesion molecules such as ICAM-1 has been reported as the consequence of long-term microgravity [28, 29]. In our study, we found that ICAM-1 surface expression responds to gravity changes in BV-2 microglial cells within 20 seconds. The rapid and reversible changes of ICAM-1 on the cell surface suggest a direct gravity-sensitive effect on the membrane compartment or on protein folding, whereas transcriptional or proteolytic processes are rather unlikely as they would be too slow. Interestingly, ICAM-1 cell surface expression in microgravity was upregulated in macrophage-like differentiated human U937 cells (Figures 4, 5, and 7) but downregulated in murine BV-2 microglial cells (Figure 2). In primary human macrophages, no clear conclusion is possible, because of the very low fluorescence levels in the analysis of parabolic flight samples (Figure 5). However, the clinostat experiments with primary human macrophages (Figure 4) suggest an upregulation of ICAM-1 in microgravity. The different ICAM-1 regulation between macrophage-like differentiated human U937 and murine BV-2 microglial cells in microgravity could be the consequence of the different species (murine and human) or different molecular and functional features of peripheral macrophages and CNS macrophages.

In our study, we detected effect of microgravity on ICAM-1 mRNA expression, neither in a parabolic flight experiment, nor during the sounding rocket experiment (Figure 7). In a previous study, also no effect of simulated microgravity on ICAM-1 mRNA expression in endothelial cells could be found [15]. However, performing pathway analyses on ICAM-1 related pathways, we identified the natural killer cell mediated cytotoxicity being influenced significantly after 20 sec of microgravity. After 6 min of microgravity this effect appeared to be reversed and we found the NF-kappa B signaling pathway and the Epstein-Barr virus infection close to significant alteration. This observation is in line with findings in astronauts after long-term space missions where latent viruses persisting in a dormant state after primary infection were reactivated [30, 31]. Therefore, we hypothesize that these two pathways may be stronger affected over a longer period of microgravity. As we were not able to find an influence on the natural killer cell mediated cytotoxicity after 6 min of microgravity, we suppose this is one of the short-term reversible processes that can recover after an adaptation phase to microgravity.

Related to the regulation of surface ICAM-1 expression, internalization and receptor recycling of ICAM-1 are highly dynamic processes [32, 33] and linked to cytoskeletal function [34, 35].

Multiple investigators have reported that this complex network of fibers is sensitive to environmental factors such as microgravity and altered gravitational forces [36–38]. Several studies demonstrated modifications of the actin and microtubule cytoskeleton in real and simulated microgravity in lymphocytes, astrocytes, neurons, glial cells, mesenchymal stem cells, and thyroid carcinoma cells [36–41]. Morphological differences of both the microtubule and actin components of the cytoskeleton have been observed in cells grown in real and simulated microgravity [39–43]. During space flight, actin reorganization in response to the gravity level and abnormal assembly of actin stress fibers has been reported [44–46].

We conclude that disturbed immune function in microgravity could be a consequence of ICAM-1 modulation in the monocyte/macrophage system, which in turn could have a strong impact on the cells' interaction with T lymphocytes and migration. An experiment under real microgravity conditions on board of the ISS was conducted by Italian and Swiss investigators to test the hypothesis that lack of interaction might be the reason for the loss of activity of T cells in microgravity [14]. The investigation consisted of analyzing the cap formation of the adhesion proteins LFA-1 on T cells and ICAM-1 on monocytes. The data showed that LFA-1/ICAM-1 interactions occur in space but are dependent on activation time; they show differences in number, arrangement, and fluorescence intensity. Thus, LFA-1 and ICAM-1 adhesion proteins seem to be sensitive to real microgravity, without being altered in their interaction. Loss of functional ICAM-1 in the brain-resident microglial cells bears the risk of a significant impairment of the CNS immune system. Indeed, reactivation and shed of varicella-zoster virus (VZV) have been reported in astronauts [30, 31], a virus which becomes latent in the nervous system after primary infection, but is reactivated frequently in immune suppressed individuals.

In conclusion, we found that ICAM-1 can be downregulated rapidly and reversibly in BV-2 microglial cells and upregulated in macrophage-like differentiated U937 cells in response to microgravity. In both cell types, long-term effects up to several days could be detected. Thus, ICAM-1 can be considered as a rapid-reacting and sustained gravity-regulated molecule in mammalian cells.

## Supplementary Material

Supplement 1: Pathway enrichment analysis. The Pathway enrichment analysis was performed using Partek Genomics Suite 6.6 and the KEGG human pathway library, P values were calculated by the Fisher exact test. Enrichment analysis was applied on the genes showing differential expression with *P* values of <0.05 and fold change >+1.5 or <−1.5.
Pathway enrichment analysis were summarized in Tables (19th DLR PFC - µg vs 1g - NATURAL KILLER CELL MEDIATED CYTOTOXICITY, TEXUS-49 - µg vs 1g - EPSTEIN-BARR VIRUS INFECTION, TEXUS-49 - µg vs 1g - NF-KAPPA B SIGNALING PATHWAY) and pathway figures. Pathway analysis revealed an influence of real microgravity on the Natural killer cell mediated cytotoxicity of monocytic U937 cells. Additionally, the NF-kappa B signaling pathway (enrichment *P*-value 0.0632651) and the Epstein-Barr virus infection (enrichment *P*-value 0.0641782) appeared sensitive to microgravity compared to baseline.

## Figures and Tables

**Table 1:** Regulation of ICAM-1 in cells of the monocyte/macrophage system in microgravity. Overview about cell types, microgravity platforms, the experiment durations, analysis methods, number of experiments, and effects on ICAM-1.

Experimental platform	Cell types	Experimental groups	Analysis method	Number of replicates	Time of *μ*g exposal	Regulation of ICAM-1	
						*μ*g/1 g	1 g/incubator control
Clinorotation	BV2 microglia cells	*μ*g group 1 g control group	FACS (20.000 events/sample)	*N* = 3	24 h	↓	
	Primary macrophages	*μ*g group 1 g control group	FACS (5.000 events/sample)	*N* = 3	1–5 d	↑	
	U937 macrophages	*μ*g group 1 g control group	FACS (10.000 events/sample)	*N* = 6	1–5 d	↑	

Parabolic flight	BV2 microglia cells	*μ*g group 1 g control group	Confocal microscopy/ random sampling module (>500 cells/sample)	*N* = 3 (w/o)		↑	
				*N* = 3 (with PMA)	20 sec	↓	
				*N* = 3 (with TNF-a)		—	
	U937 macrophages	*μ*g group 1 g control group Ground control	FACS (10.000 events/sample)	*N* = 9	20 sec	↑	↓
				*N* = 9			
				*N* = 6			
	U937 monocytes	*μ*g group 1 g control group Ground control	FACS (10.000 events/sample)	*N* = 9 (w/o PMA)	20 sec	—	—
				*N* = 5 (with PMA)			
				*N* = 12 (w/o PMA)			
				*N* = 6 (with PMA)			
				*N* = 2 (w/o PMA)			
				*N* = 1 (with PMA)			
		*μ*g group 1 g control group 1.8 g control group Hardware control	Microarray	*N* = 8	20 sec	—	—
				*N* = 8			
				*N* = 6			
				*N* = 6			
	Primary macrophages	*μ*g group 1 g control group	FACS (10.000 events/sample)	*N* = 9	20 sec	—	
				*N* = 9			

SIMBOX Shenzhou 8	U937 Macrophages	*μ*g group 1 g control group Incubator control	Confocal microscopy (300 cells/sample) (650 cells/sample) (1000 cells/sample)	*N* = 1	5 d	↑	↓

TEXUS-49	U937 monocytes	*μ*g group baseline group Hardware control	Microarray	*N* = 7	10 min	—	—
				*N* = 5			
				*N* = 6			

## Abbreviations

BL: Baseline
CC: Cell culture control
DLR: German Aerospace Center
ESA: European Space Agency
ESRANGE: European Space and Sounding Rocket Range
EUE: Experiment Unique Equipment
FACS: Fluorescence activated cell sorting
FCS: Fetal calf serum
GC: Ground control
H/W: Hardware
ICAM-1: Intercellular adhesion molecule 1
JSLC: Jiuquan Satellite Launch Center
LFA-1: Lymphocyte function-associated antigen 1
MAC-1: Myelomonocytic leukocyte integrin CD11b
*μ*g: Microgravity
MMS: Monocyte-macrophage-system
PBMC: Peripheral blood mononuclear cell
PEEK: Polyether ether ketone
PFA: Paraformaldehyde
PFC: Parabolic flight campaign
PITC: Payload integration and test center
PMA: 12-O-Tetradecanoylphorbol-13-acetate
RCCS: Rotary Cell Culture Systems
RFI: Relative fluorescence intensity
RIN: RNA integrity number
RPM: Random positioning machine
SSC: Swedish Space Corporation
SIMBOX: Science in Microgravity Box
TUNEL: TdT-mediated dUTP-biotin nick end labeling.
