# Supplementary material for: Regulation of ICAM-1 in Cells of the Monocyte/Macrophage System in Microgravity
Source: Biomed Res Int. 2015 Jan 13;2015:538786. doi: 10.1155/2015/538786 (PMC4309248; doi:10.1155/2015/538786)
Supplement: Supplementary file 1 — Supplement 1: Pathway enrichment analysis. The Pathway enrichment analysis was performed using Partek Genomics Suite 6.6 and the KEGG human pathway library, P values were calculated by the Fisher exact test. Enrichment analysis was applied on the genes showing differential expression with P values of <0.05 and fold change >+1.5 or <−1.5. Pathway enrichment analysis were summarized in Tables (19th DLR PFC - µg vs 1g - NATURAL KILLER CELL MEDIATED CYTOTOXICITY, TEXUS-49 - µg vs 1g - EPSTEIN-BARR VIRUS INFECTION, TEXUS-49 - µg vs 1g - NF-KAPPA B SIGNALING PATHWAY) and pathway figures. Pathway analysis revealed an influence of real microgravity on the Natural killer cell mediated cytotoxicity of monocytic U937 cells. Additionally, the NF-kappa B signaling pathway (enrichment P-value 0.0632651) and the Epstein-Barr virus infection (enrichment P-value 0.0641782) appeared sensitive to microgravity compared to baseline. [file 538786.f1.zip › Supplement 1_ pathway figures.pdf]

NF-KAPPA B SIGNALING PATHWAY

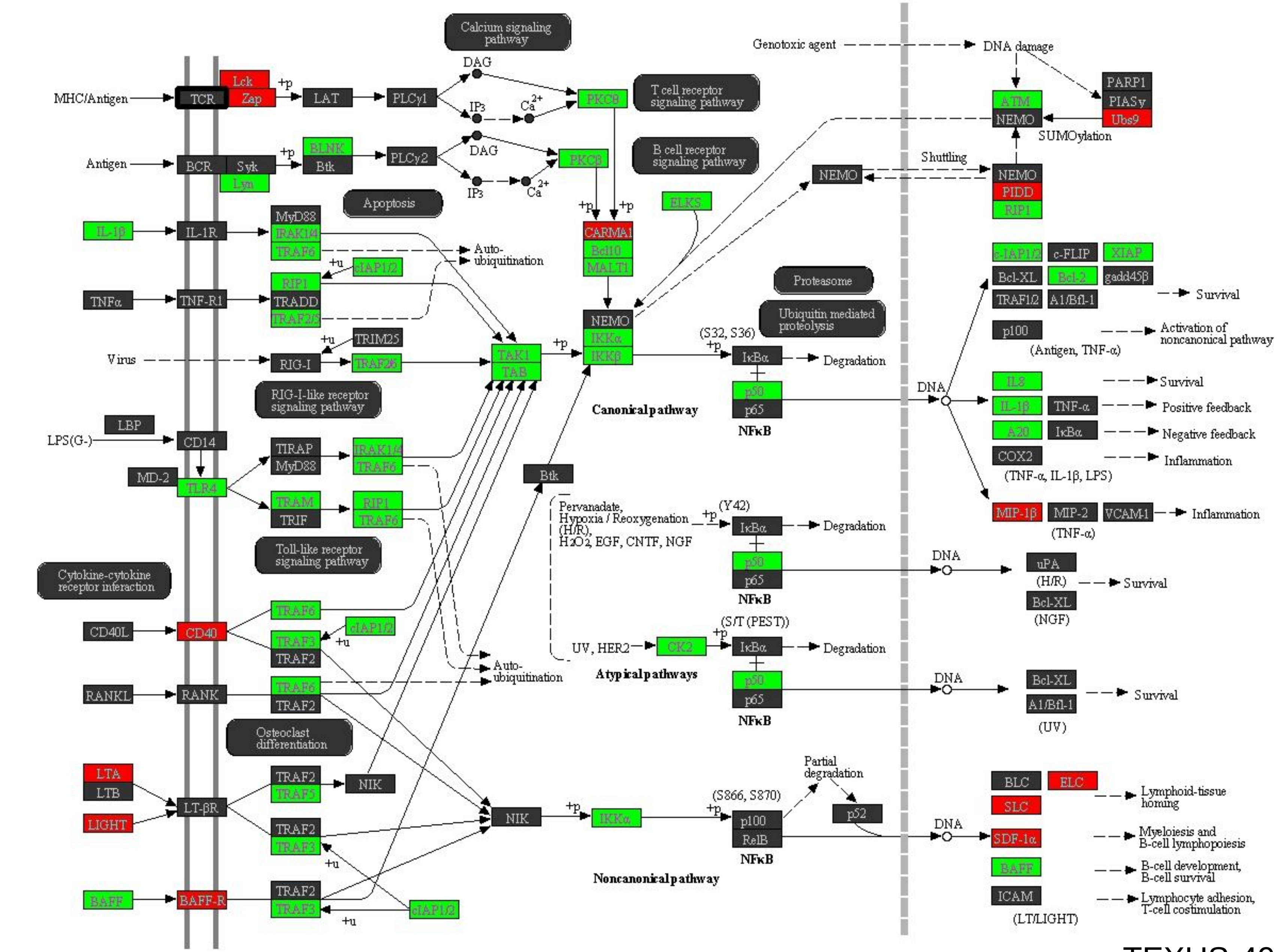

04064 5/30/13
(c) Kanehisa Laboratories

TEXUS-49

EPSTEIN-BARR VIRUS INFECTION

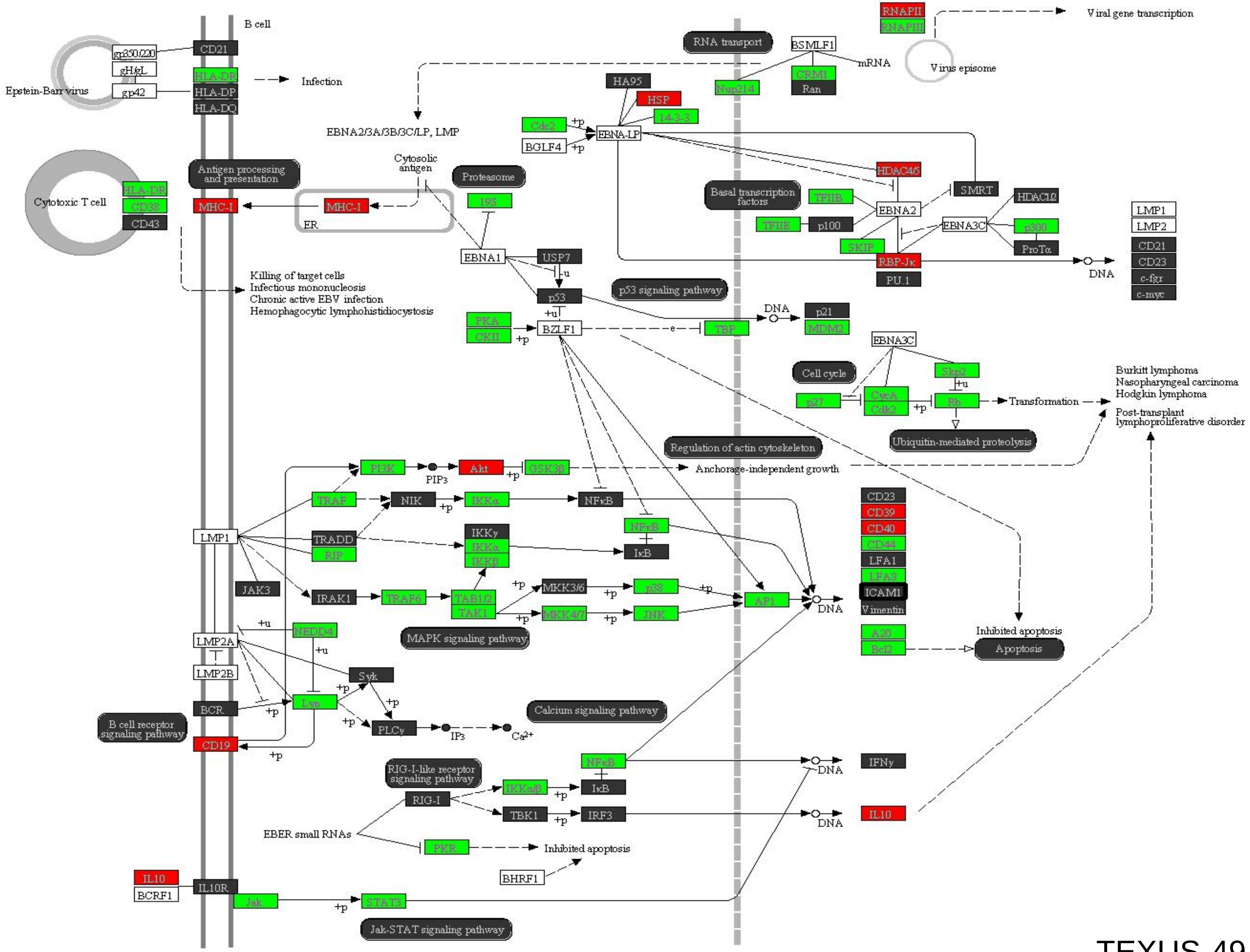

TEXUS-49

05169 5/30/13

(c) Kanehisa Laboratories

NATURAL KILLER CELL MEDIATED CYTOTOXICITY

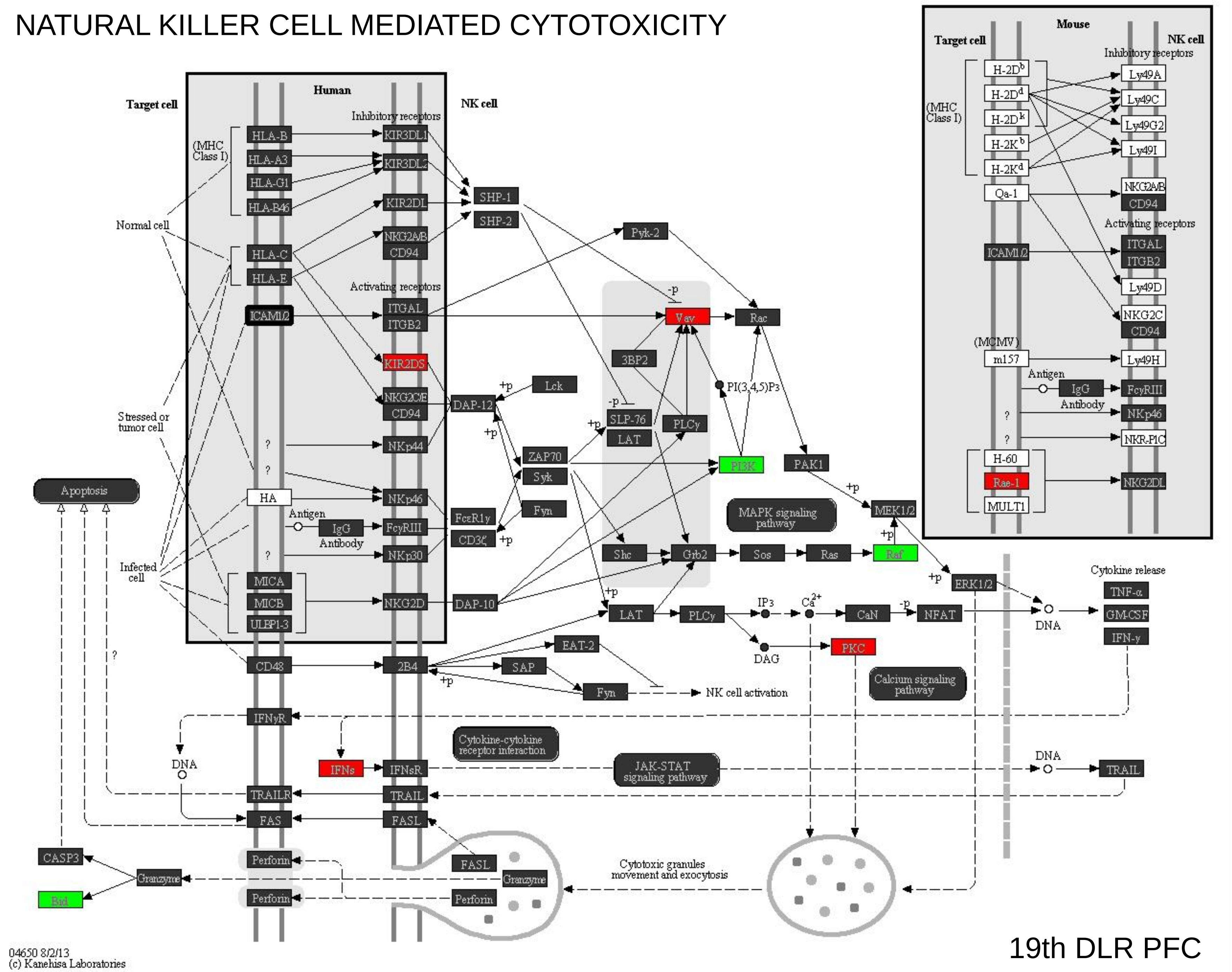

19th DLR PFC
