# Supplementary material for: Regulation of ICAM-1 in Cells of the Monocyte/Macrophage System in Microgravity
Source: Biomed Res Int. 2015 Jan 13;2015:538786. doi: 10.1155/2015/538786 (PMC4309248; doi:10.1155/2015/538786)
Supplement: Supplementary file 1 — Supplement 1: Pathway enrichment analysis. The Pathway enrichment analysis was performed using Partek Genomics Suite 6.6 and the KEGG human pathway library, P values were calculated by the Fisher exact test. Enrichment analysis was applied on the genes showing differential expression with P values of <0.05 and fold change >+1.5 or <−1.5. Pathway enrichment analysis were summarized in Tables (19th DLR PFC - µg vs 1g - NATURAL KILLER CELL MEDIATED CYTOTOXICITY, TEXUS-49 - µg vs 1g - EPSTEIN-BARR VIRUS INFECTION, TEXUS-49 - µg vs 1g - NF-KAPPA B SIGNALING PATHWAY) and pathway figures. Pathway analysis revealed an influence of real microgravity on the Natural killer cell mediated cytotoxicity of monocytic U937 cells. Additionally, the NF-kappa B signaling pathway (enrichment P-value 0.0632651) and the Epstein-Barr virus infection (enrichment P-value 0.0641782) appeared sensitive to microgravity compared to baseline. [file 538786.f1.zip › Tabelle_19th DLR PFC - μg vs 1g - NATURAL KILLER CELL MEDIATED CYTOTOXICITY-rev.pdf]

19th DLR PFC - µg vs 1g - NATURAL KILLER CELL MEDIATED CYTOTOXICITY					
**Gene Symbol**	**Gene name**	**Transcript ID**	**p-value**	**Fold-Change**	**Up / down-regulation**
BID	BH3 interacting domain death agonist	BC009197	0.023	-1.570	µg down vs 1g
BRAF	v-raf murine sarcoma viral oncogene homolog B1	NM_004333	0.033	-1.607	µg down vs 1g
IFNA6	interferon, alpha 6	NM_021002	0.048	1.741	µg up vs 1g
IFNA14	interferon, alpha 14	NM_002172	0.046	1.632	µg up vs 1g
KIR2DS2	killer cell immunoglobulin-like receptor, two domains, short cytoplasmic tail, 2	AJ002104	0.049	1.869	µg up vs 1g
PIK3R1	phosphoinositide-3-kinase, regulatory subunit 1 (p85 alpha)	BC094795	0.034	-1.839	µg down vs 1g
PIK3R3	phosphoinositide-3-kinase, regulatory subunit 3 (p55, gamma)	AF028785	0.037	-1.595	µg down vs 1g
PRKCG	protein kinase C, gamma	BC047876	0.027	1.542	µg up vs 1g
RAET1E	retinoic acid early transcript 1E	BC101976	0.042	1.502	µg up vs 1g
VAV2	vav 2 oncogene	AK130475	0.033	1.606	µg up vs 1g
