# Supplementary material for: Regulation of ICAM-1 in Cells of the Monocyte/Macrophage System in Microgravity
Source: Biomed Res Int. 2015 Jan 13;2015:538786. doi: 10.1155/2015/538786 (PMC4309248; doi:10.1155/2015/538786)
Supplement: Supplementary file 1 — Supplement 1: Pathway enrichment analysis. The Pathway enrichment analysis was performed using Partek Genomics Suite 6.6 and the KEGG human pathway library, P values were calculated by the Fisher exact test. Enrichment analysis was applied on the genes showing differential expression with P values of <0.05 and fold change >+1.5 or <−1.5. Pathway enrichment analysis were summarized in Tables (19th DLR PFC - µg vs 1g - NATURAL KILLER CELL MEDIATED CYTOTOXICITY, TEXUS-49 - µg vs 1g - EPSTEIN-BARR VIRUS INFECTION, TEXUS-49 - µg vs 1g - NF-KAPPA B SIGNALING PATHWAY) and pathway figures. Pathway analysis revealed an influence of real microgravity on the Natural killer cell mediated cytotoxicity of monocytic U937 cells. Additionally, the NF-kappa B signaling pathway (enrichment P-value 0.0632651) and the Epstein-Barr virus infection (enrichment P-value 0.0641782) appeared sensitive to microgravity compared to baseline. [file 538786.f1.zip › Tabelle_TEXUS-49 - μg vs 1g - EPSTEIN-BARR VIRUS INFECTION.pdf]

TEXUS-49 - µg vs 1g - EPSTEIN-BARR VIRUS INFECTION					
**Gene Symbol**	**Gene name**	**Transcript ID**	**p-value**	**Fold-Change**	**Up / down-regulation**
AKT1	v-akt murine thymoma viral oncogene homolog 1	BX647722	0.023	1.657	µg up vs 1g
ATF2	activating transcription factor 2	NM_001880	0.018	-2.558	µg down vs 1g
BCL2	B-cell CLL/lymphoma 2	NM_000657	0.048	1.594	µg up vs 1g
BST1	bone marrow stromal cell antigen 1	NM_004334	0.003	-2.615	µg down vs 1g
CCNA2	cyclin A2	NM_001237	0.006	-2.078	µg down vs 1g
CD19	CD19 molecule	NM_001770	0.044	1.743	µg up vs 1g
CD38	CD38 molecule	BC007964	0.041	-1.646	µg down vs 1g
CD40	CD40 molecule, TNF receptor superfamily member 5	NM_001250	0.033	1.932	µg up vs 1g
CD44	CD44 molecule (Indian blood group)	NM_000610	0.004	-2.564	µg down vs 1g
CD58	CD58 molecule	X06296	0.007	-3.963	µg down vs 1g
CDK1	cyclin-dependent kinase 1	NM_033379	0.002	-3.012	µg down vs 1g
CDK2	cyclin-dependent kinase 2	NM_001798	0.013	-1.580	µg down vs 1g
CDKN1B	cyclin-dependent kinase inhibitor 1B (p27, Kip1)	NM_004064	0.001	-3.506	µg down vs 1g
CHUK	conserved helix-loop-helix ubiquitous kinase	NM_001278	0.006	-3.046	µg down vs 1g
CREBBP	CREB binding protein (Rubinstein-Taybi syndrome)	NM_004380	0.030	-2.282	µg down vs 1g
CSNK2A1	casein kinase 2, alpha 1 polypeptide	NM_001895	0.024	-1.530	µg down vs 1g
EIF2AK2	eukaryotic translation initiation factor 2-alpha kinase 2	BC093676	0.004	-1.975	µg down vs 1g
EIF2AK4	eukaryotic translation initiation factor 2 alpha kinase 4	NM_001013 703	0.006	-2.850	µg down vs 1g
EMB	embigin homolog (mouse)	NM_198449	0.013	-3.546	µg down vs 1g
ENTPD8	ectonucleoside triphosphate diphosphohydrolase 8	NM_198585	0.021	1.805	µg up vs 1g
EP300	E1A binding protein p300	NM_001429	0.044	-1.658	µg down vs 1g
GSK3B	glycogen synthase kinase 3 beta	NM_002093	0.009	-1.654	µg down vs 1g
GTF2B	general transcription factor IIB	NM_001514	0.010	-1.664	µg down vs 1g
GTF2E1	general transcription factor IIE, polypeptide 1, alpha 56kDa	NM_005513	0.006	-2.506	µg down vs 1g
GTF2E2	general transcription factor IIE, polypeptide 2, beta 34kDa	NM_002095	0.004	-2.199	µg down vs 1g

Gene Symbol	Gene name	Transcript ID	p-value	Fold-Change	Up / down-regulation
HDAC5	histone deacetylase 5	NM_001015053	0.039	1.654	µg up vs 1g
HLA-A	major histocompatibility complex, class I, A	AK125608	0.028	1.624	µg up vs 1g
HLA-DRB1	major histocompatibility complex, class II, DR beta 1	AB036991	0.002	-2.135	µg down vs 1g
HSPB2	heat shock 27kDa protein 2	NM_001541	0.016	2.081	µg up vs 1g
IKBKB	inhibitor of kappa light polypeptide gene enhancer in B-cells, kinase beta	NM_001556	0.030	-1.608	µg down vs 1g
IL10	interleukin 10	BC104252	0.042	1.540	µg up vs 1g
JAK1	Janus kinase 1 (a protein tyrosine kinase)	AB209057	0.018	-1.637	µg down vs 1g
LYN	v-yes-1 Yamaguchi sarcoma viral related oncogene homolog	NM_002350	0.002	-1.640	µg down vs 1g
MAP2K4	mitogen-activated protein kinase kinase 4	NM_003010	0.027	-2.140	µg down vs 1g
MAP3K7	mitogen-activated protein kinase kinase kinase 7	NM_145331	0.014	-2.325	µg down vs 1g
MAPK8	mitogen-activated protein kinase 8	NM_139047	0.006	-3.835	µg down vs 1g
MAPK9	mitogen-activated protein kinase 9	NM_002752	0.011	-3.423	µg down vs 1g
MAPK14	mitogen-activated protein kinase 14	NM_139013	0.015	-1.635	µg down vs 1g
MDM2	Mdm2, transformed 3T3 cell double minute 2, p53 binding protein (mouse)	NM_006881	0.043	-2.874	µg down vs 1g
NEDD4	neural precursor cell expressed, developmentally down-regulated 4	NM_198400	0.018	-2.883	µg down vs 1g
NFKB1	nuclear factor of kappa light polypeptide gene enhancer in B-cells 1 (p105)	NM_003998	0.018	-1.843	µg down vs 1g
NUP214	nucleoporin 214kDa	NM_005085	0.041	-1.569	µg down vs 1g
PIK3CA	phosphoinositide-3-kinase, catalytic, alpha polypeptide	NM_006218	0.019	-3.460	µg down vs 1g
PIK3CB	phosphoinositide-3-kinase, catalytic, beta polypeptide	NM_006219	0.008	-3.202	µg down vs 1g
PIK3CG	phosphoinositide-3-kinase, catalytic, gamma polypeptide	NM_002649	0.015	-4.849	µg down vs 1g
PIK3R1	phosphoinositide-3-kinase, regulatory subunit 1 (p85 alpha)	NM_181504	0.019	-2.418	µg down vs 1g
PIK3R3	phosphoinositide-3-kinase, regulatory subunit 3 (p55, gamma)	NM_003629	0.021	-2.685	µg down vs 1g
POLR2B	polymerase (RNA) II (DNA directed) polypeptide B, 140kDa	NM_000938	0.014	-1.690	µg down vs 1g
POLR2D	polymerase (RNA) II (DNA directed) polypeptide D	BC093795	0.011	-1.520	µg down vs 1g
POLR2H	polymerase (RNA) II (DNA directed) polypeptide H	NM_006232	0.003	-1.615	µg down vs 1g

Gene Symbol	Gene name	Transcript ID	p-value	Fold-Change	Up / down-regulation
POLR2J2	DNA directed RNA polymerase II polypeptide J-related gene	NM_145325	0.024	1.505	µg up vs 1g
POLR2K	polymerase (RNA) II (DNA directed) polypeptide K, 7.0kDa	BC018157	0.011	-1.628	µg down vs 1g
POLR3A	polymerase (RNA) III (DNA directed) polypeptide A, 155kDa	BC014399	0.038	-2.005	µg down vs 1g
POLR3B	polymerase (RNA) III (DNA directed) polypeptide B	NM_018082	0.018	-2.797	µg down vs 1g
POLR3C	polymerase (RNA) III (DNA directed) polypeptide C (62kD)	NM_006468	0.004	-1.514	µg down vs 1g
POLR3E	polymerase (RNA) III (DNA directed) polypeptide E (80kD)	NM_018119	0.018	-1.559	µg down vs 1g
POLR3F	polymerase (RNA) III (DNA directed) polypeptide F, 39 kDa	NM_006466	0.035	-3.186	µg down vs 1g
POLR3GL	polymerase (RNA) III (DNA directed) polypeptide G (32kD) like	NM_032305	0.005	-1.846	µg down vs 1g
PRKACB	protein kinase, cAMP-dependent, catalytic, beta	NM_207578	0.022	-4.445	µg down vs 1g
PRKACG	protein kinase, cAMP-dependent, catalytic, gamma	NM_002732	0.034	1.620	µg up vs 1g
PRKX	protein kinase, X-linked	BC041073	0.016	-2.500	µg down vs 1g
PSMC6	proteasome (prosome, macropain) 26S subunit, ATPase, 6	NM_002806	0.003	-2.377	µg down vs 1g
PSMD1	proteasome (prosome, macropain) 26S subunit, non-ATPase, 1	NM_002807	0.003	-2.305	µg down vs 1g
PSMD6	proteasome (prosome, macropain) 26S subunit, non-ATPase, 6	NM_014814	0.007	-1.542	µg down vs 1g
PSMD12	proteasome (prosome, macropain) 26S subunit, non-ATPase, 12	NM_002816	0.003	-3.215	µg down vs 1g
PSMD14	proteasome (prosome, macropain) 26S subunit, non-ATPase, 14	NM_005805	0.014	-1.753	µg down vs 1g
RB1	retinoblastoma 1 (including osteosarcoma)	NM_000321	0.001	-2.945	µg down vs 1g
RBPJ	recombination signal binding protein for immunoglobulin kappa J region	BC020780	0.007	-1.581	µg down vs 1g
RBPJL	recombining binding protein suppressor of hairless (Drosophila)-like	AB024964	0.048	1.686	µg up vs 1g
RIPK1	receptor (TNFRSF)-interacting serine-threonine kinase 1	NM_003804	0.034	-1.864	µg down vs 1g
RPN2	ribophorin II	BC020222	0.010	-1.629	µg down vs 1g
SHFM1	split hand/foot malformation (ectrodactyly) type 1	NM_006304	0.004	-2.457	µg down vs 1g
SKP2	S-phase kinase-associated protein 2 (p45)	NM_005983	0.015	-1.677	µg down vs 1g
SNW1	SNW domain containing 1	NM_012245	0.004	-1.672	µg down vs 1g
STAT3	signal transducer and activator of transcription 3 (acute-phase response factor)	NM_213662	0.022	-2.066	µg down vs 1g

Gene Symbol	Gene name	Transcript ID	p-value	Fold-Change	Up / down-regulation
TAB2	TGF-beta activated kinase 1/MAP3K7 binding protein 2	BC035910	0.015	-3.617	µg down vs 1g
TBPL1	TBP-like 1	NM_004865	0.006	-2.211	µg down vs 1g
TNFAIP3	tumor necrosis factor, alpha-induced protein 3	BC114480	0.009	-2.410	µg down vs 1g
TRAF3	TNF receptor-associated factor 3	NM_003300	0.001	-1.512	µg down vs 1g
TRAF5	TNF receptor-associated factor 5	NM_001033910	0.033	-1.551	µg down vs 1g
TRAF6	TNF receptor-associated factor 6	NM_004620	0.037	-2.093	µg down vs 1g
XPO1	exportin 1 (CRM1 homolog, yeast)	NM_003400	0.002	-1.997	µg down vs 1g
YWHAB	tyrosine 3-monooxygenase/tryptophan 5-monooxygenase activation protein, beta polypeptid	BC001359	0.003	-1.722	µg down vs 1g
YWHAG	tyrosine 3-monooxygenase/tryptophan 5-monooxygenase activation protein, gamma polypept	NM_012479	0.009	-2.206	µg down vs 1g
YWHAH	tyrosine 3-monooxygenase/tryptophan 5-monooxygenase activation protein, eta polypeptid	NM_003405	0.015	-1.669	µg down vs 1g
YWHAZ	tyrosine 3-monooxygenase/tryptophan 5-monooxygenase activation protein, zeta polypeptid	NM_003406	0.002	-1.647	µg down vs 1g
