# Supplementary material for: Regulation of ICAM-1 in Cells of the Monocyte/Macrophage System in Microgravity
Source: Biomed Res Int. 2015 Jan 13;2015:538786. doi: 10.1155/2015/538786 (PMC4309248; doi:10.1155/2015/538786)
Supplement: Supplementary file 1 — Supplement 1: Pathway enrichment analysis. The Pathway enrichment analysis was performed using Partek Genomics Suite 6.6 and the KEGG human pathway library, P values were calculated by the Fisher exact test. Enrichment analysis was applied on the genes showing differential expression with P values of <0.05 and fold change >+1.5 or <−1.5. Pathway enrichment analysis were summarized in Tables (19th DLR PFC - µg vs 1g - NATURAL KILLER CELL MEDIATED CYTOTOXICITY, TEXUS-49 - µg vs 1g - EPSTEIN-BARR VIRUS INFECTION, TEXUS-49 - µg vs 1g - NF-KAPPA B SIGNALING PATHWAY) and pathway figures. Pathway analysis revealed an influence of real microgravity on the Natural killer cell mediated cytotoxicity of monocytic U937 cells. Additionally, the NF-kappa B signaling pathway (enrichment P-value 0.0632651) and the Epstein-Barr virus infection (enrichment P-value 0.0641782) appeared sensitive to microgravity compared to baseline. [file 538786.f1.zip › Tabelle_TEXUS-49 - μg vs 1g - NF-KAPPA B SIGNALING PATHWAY.pdf]

TEXUS-49 - µg vs 1g - NF-KAPPA B SIGNALING PATHWAY					
**Gene Symbol**	**Gene name**	**Transcript ID**	**p-value**	**Fold-Change**	**Up / down-regulation**
ATM	ataxia telangiectasia mutated (includes complementation groups A, C and D)	NM_000051	0.021	-2.239	µg down vs 1g
BCL2	B-cell CLL/lymphoma 2	NM_000657	0.048	1.594	µg up vs 1g
BCL10	B-cell CLL/lymphoma 10	BC053617	0.009	-2.443	µg down vs 1g
BIRC2	baculoviral IAP repeat-containing 2	NM_001166	0.008	-4.046	µg down vs 1g
BLNK	B-cell linker	NM_013314	0.015	-1.836	µg down vs 1g
CARD11	caspase recruitment domain family, member 11	NM_032415	0.015	4.774	µg up vs 1g
CCL4L2	chemokine (C-C motif) ligand 4-like 2	AY766457	0.042	1.816	µg up vs 1g
CCL19	chemokine (C-C motif) ligand 19	NM_006274	0.016	1.782	µg up vs 1g
CCL21	chemokine (C-C motif) ligand 21	BC027918	0.038	1.627	µg up vs 1g
CD40	CD40 molecule, TNF receptor superfamily member 5	NM_001250	0.033	1.932	µg up vs 1g
CHUK	conserved helix-loop-helix ubiquitous kinase	NM_001278	0.006	-3.046	µg down vs 1g
CSNK2A1	casein kinase 2, alpha 1 polypeptide	NM_001895	0.024	-1.530	µg down vs 1g
CXCL12	chemokine (C-X-C motif) ligand 12 (stromal cell-derived factor 1)	NM_001033 886	0.009	2.159	µg up vs 1g
ERC1/RAB6IP2	RAB6 interacting protein 2	NM_015064	0.045	-1.878	µg down vs 1g
IKBKB	inhibitor of kappa light polypeptide gene enhancer in B-cells, kinase beta	NM_001556	0.030	-1.608	µg down vs 1g
IL1B	interleukin 1, beta	NM_000576	0.014	-1.538	µg down vs 1g
IL8	interleukin 8	NM_000584	0.006	-3.175	µg down vs 1g
IRAK4	interleukin-1 receptor-associated kinase 4	NM_016123	0.012	-2.498	µg down vs 1g
LCK	lymphocyte-specific protein tyrosine kinase	U23852	0.043	3.615	µg up vs 1g
LTA	lymphotoxin alpha (TNF superfamily, member 1)	DQ123822	0.009	2.031	µg up vs 1g
LYN	v-yes-1 Yamaguchi sarcoma viral related oncogene homolog	NM_002350	0.002	-1.640	µg down vs 1g
MAL	mal, T-cell differentiation protein	BC003006	0.032	2.990	µg up vs 1g
MALT1	mucosa associated lymphoid tissue lymphoma translocation gene 1	BC030143	0.012	-3.701	µg down vs 1g
MAP3K7	mitogen-activated protein kinase kinase kinase 7	NM_145331	0.014	-2.325	µg down vs 1g

Gene Symbol	Gene name	Transcript ID	p-value	Fold-Change	Up / down-regulation
NFKB1	nuclear factor of kappa light polypeptide gene enhancer in B-cells 1 (p105)	NM_003998	0.018	-1.843	µg down vs 1g
PIDD/LRDD	leucine-rich repeats and death domain containing	NM_018494	0.024	1.662	µg up vs 1g
PRKCB	protein kinase C, beta 1	BC036472	0.003	-1.624	µg down vs 1g
PRKCQ	protein kinase C, theta	NM_006257	0.009	-1.905	µg down vs 1g
RIPK1	receptor (TNFRSF)-interacting serine-threonine kinase 1	NM_003804	0.034	-1.864	µg down vs 1g
TAB2	mitogen-activated protein kinase kinase kinase 7 interacting protein 2	BC035910	0.015	-3.617	µg down vs 1g
TICAM2	toll-like receptor adaptor molecule 2	NM_021649	0.024	-2.373	µg down vs 1g
TLR4	toll-like receptor 4	NM_138557	0.024	-3.937	µg down vs 1g
TNFAIP3	tumor necrosis factor, alpha-induced protein 3	BC114480	0.009	-2.410	µg down vs 1g
TNFRSF13C	tumor necrosis factor receptor superfamily, member 13C	NM_052945	0.015	1.987	µg up vs 1g
TNFSF13B	tumor necrosis factor (ligand) superfamily, member 13b	BC020674	0.019	-1.768	µg down vs 1g
TNFSF14	tumor necrosis factor (ligand) superfamily, member 14	NM_003807	0.014	1.522	µg up vs 1g
TRAF3	TNF receptor-associated factor 3	NM_003300	0.001	-1.512	µg down vs 1g
TRAF5	TNF receptor-associated factor 5	NM_001033910	0.033	-1.551	µg down vs 1g
TRAF6	TNF receptor-associated factor 6	NM_004620	0.037	-2.093	µg down vs 1g
UBE2I	ubiquitin-conjugating enzyme E2I (UBC9 homolog, yeast)	BC000744	0.014	1.977	µg up vs 1g
XIAP	baculoviral IAP repeat-containing 4	BC032729	0.039	-2.683	µg down vs 1g
ZAP70	zeta-chain (TCR) associated protein kinase 70kDa	NM_207519	0.032	1.690	µg up vs 1g
